# Soil depth matters: shift in composition and inter-kingdom co-occurrence patterns of microorganisms in forest soils

**DOI:** 10.1093/femsec/fiab022

**Published:** 2021-02-06

**Authors:** Sunil Mundra, O Janne Kjønaas, Luis N Morgado, Anders Kristian Krabberød, Yngvild Ransedokken, Håvard Kauserud

**Affiliations:** Section for Genetics and Evolutionary Biology (EvoGene), Department of Biosciences, University of Oslo, NO-0316 Oslo, Norway; Department of Biology, College of Science, United Arab Emirates University, Al-Ain, Abu-Dhabi, UAE; NIBIO, Department of Terrestrial Ecology, NO-1431 Ås, Norway; Section for Genetics and Evolutionary Biology (EvoGene), Department of Biosciences, University of Oslo, NO-0316 Oslo, Norway; Naturalis Biodiversity Center, 2300 RA Leiden, the Netherlands; Section for Genetics and Evolutionary Biology (EvoGene), Department of Biosciences, University of Oslo, NO-0316 Oslo, Norway; Faculty of Environmental and Natural Resource Management, Norwegian University of Life Sciences, NO-1432 Ås, Norway; Section for Genetics and Evolutionary Biology (EvoGene), Department of Biosciences, University of Oslo, NO-0316 Oslo, Norway

**Keywords:** co-occurrences patterns, metabarcoding, microbial communities, microbial interactions, *Betula pubescens*, boreal birch forest

## Abstract

Soil depth represents a strong physiochemical gradient that greatly affects soil-dwelling microorganisms. Fungal communities are typically structured by soil depth, but how other microorganisms are structured is less known. Here, we tested whether depth-dependent variation in soil chemistry affects the distribution and co-occurrence patterns of soil microbial communities. This was investigated by DNA metabarcoding in conjunction with network analyses of bacteria, fungi, as well as other micro-eukaryotes, sampled in four different soil depths in Norwegian birch forests. Strong compositional turnover in microbial assemblages with soil depth was detected for all organismal groups. Significantly greater microbial diversity and fungal biomass appeared in the nutrient-rich organic layer, with sharp decrease towards the less nutrient-rich mineral zones. The proportions of copiotrophic bacteria, Arthropoda and Apicomplexa were markedly higher in the organic layer, while patterns were opposite for oligotrophic bacteria, Cercozoa, Ascomycota and ectomycorrhizal fungi. Network analyses indicated more intensive inter-kingdom co-occurrence patterns in the upper mineral layer (0–5 cm) compared to the above organic and the lower mineral soil, signifying substantial influence of soil depth on biotic interactions. This study supports the view that different microbial groups are adapted to different forest soil strata, with varying level of interactions along the depth gradient.

## Teaser

This study highlights the importance of different soil horizons as well as a combination of markers when assessing microorganisms in forest soil and provide hypotheses about depth-dependent biotic interactions.

## INTRODUCTION

Forest soils harbour diverse prokaryotic and eukaryotic microbial assemblages that are crucial for overall ecosystem functioning (Bardgett and van der Putten 2014). Bacteria and fungi are the primary organisms controlling litter decomposition. The filamentous growth and extracellular enzymes secretion ability makes fungi well suited for recalcitrant soil organic matter (SOM) and cellulose decomposition (Lindahl, Taylor and Finlay 2002; Baldrian and Valášková 2008; Bödeker *et al*. 2014). Other fungi form mutualistic association with plant roots (mycorrhiza), which improves plant access to soil nutrients, and in exchange, they gain carbohydrates from their host plants. Fungi perform a range of functions in the soil and link C and nutrient flow between primary producers and higher trophic levels, making them important players in the microbial food webs (Geisen and Bonkowski 2018). Bacteria preferentially utilise low molecular mass organic compounds produced by biopolymer decomposition via fungi (Štursová *et al*. 2012). However, bacteria are also able to decompose cellulose and other plant polysaccharides (López-Mondéjar *et al*. 2016) and study even suggests they are able to decompose lignin (Wilhelm *et al*. 2018). Micro-eukaryotes other than fungi have long been neglected in surveys of forest ecosystems and barely studied in the topsoil (Geisen *et al*. 2017; Mahé *et al*. 2017; Venter, Nitsche and Arndt 2018; Oliverio *et al*. 2020). This polyphyletic group of organisms is extremely diverse with both multicellular (e.g. invertebrates) and unicellular (e.g. ciliates, cercozoans, apicomplexans and protists) life forms. Protists are among the main consumers of soil bacteria and fungi (Geisen *et al*. 2017; Seppey *et al*. 2017). Protists are also predators of soil invertebrates and their diversity correlates with diversity of their hosts (Singer *et al*. 2020). They form a dynamic hub in the soil microbiome, exerting top-down control over bacterial and fungal populations (Crowther *et al*. 2013; Xiong *et al*. 2017). However, we lack comprehensive studies analysing the co-occurrence patterns of all three different organismal groups (bacteria, fungi and micro-eukaryotes) from the same soil niche.

A wide range of edaphic factors can shape the composition of forest soil microbial communities, such as soil pH and nutrients, quantity and quality of litter input, as well as root-derived C (Baldrian 2017). Since nearly all of these factors vary with soil depth, a corresponding shift in the microbial communities is expected. Considering that pH is the major driver for bacterial richness distribution in soil (Fierer and Jackson 2006; Rousk *et al*. 2010), we also expect sharp change in bacterial richness with depth, where soil pH changes. Indeed, previous studies have shown depth related trends in fungal communities both at species level (Rosling *et al*. 2003; Baldrian *et al*. 2012) and functional level (Žifčáková *et al*. 2017) in coniferous forests. Further, in a reciprocal transplantation experiment, Bödeker *et al*. (2016) revealed that competition between saprotrophic and ectomycorrhizal fungi was important for regulating their vertical distribution. Depth-dependent structure in fungal communities has previously been observed in both spruce and beech forest from Norway (Asplund *et al*. 2018). A decrease in diversity, biomass and fungal enzyme activity with increasing depth, as well as higher gene transcription activity in the litter horizon of coniferous forest, have also been reported (Baldrian *et al*. 2013; Voříšková *et al*. 2014; Žifčáková *et al*. 2017). Saprotrophic fungi largely colonize the upper litter and poorly decomposed organic matters due to their ability to utilise recalcitrant plant residues (Lindahl *et al*. 2007; Voříšková *et al*. 2014). As the proportion of organic matter decreases with depth, the abundance of saprotrophic fungi decreases. Ectomycorrhizal (ECM) fungi, on the other hand, which are suggested to be key players in forest C dynamics (Clemmensen *et al*. 2013), increase in the lower humus and mineral soil (Dickie, Xu and Koide 2002). The newly discovered Archaeorhizomycetes, lacking typical mycorrhizal structures but possessing decomposition ability, may dominate in the deeper soils of coniferous forests (Rosling *et al*. 2011). Most studies both in coniferous and deciduous forest focus on the litter layer and the upper organic soil horizon (Hartmann *et al*. 2012; Tedersoo *et al*. 2014; Bahram *et al*. 2018), but there is evidence for that the deeper mineral soils needs to be included to assess the overall microbial diversity (Rosling *et al*. 2003; Lindahl *et al*. 2007; Jumpponen, Jones and Blair 2010; Santalahti *et al*. 2016; Du *et al*. 2017). Compared to fungi, there are few studies addressing the vertical distribution of other micro-eukaryotes (Ekelund, Rønn and Christensen 2001; Potapov *et al*. 2017) and bacteria (Baldrian *et al*. 2012; Eilers *et al*. 2012; Hartmann *et al*. 2012; López-Mondéjar *et al*. 2015; Du *et al*. 2017; Pereira *et al*. 2017) in forest soils, and their distribution have rarely been analyzed through the mineral soil profile (< 30 cm).

Inter-kingdom co-occurrence patterns of soil microbes has been shown to vary depending on soil types, organic C and pH level (Creamer *et al*. 2016; Xiong *et al*. 2017; de Araujo *et al*. 2018), and are tightly linked with above-ground vegetation (Wardle *et al*. 2004). Recently, Hernandez *et al*. (2021) demonstrated that in scrub habitats, under stressful environment conditions microbial community network destabilizes by decreasing modularity as well as negative: positive cohesion. Results from co-occurrence network-based analyses does not necessarily reflect biotic interactions. However, such analyses allows us to generate hypotheses about potential biotic associations among community members and enables us to examine cross-kingdom relationships in large microbial community data sets generated in high-throughput sequencing (HTS) studies of environmental DNA (de Vries *et al*. 2018). By employing network analyses for soil communities we can produce hypotheses about (i) the functional roles of uncultured microorganisms (Fuhrman and Steele 2008; Chaffron *et al*. 2010); (ii) niche spaces shared by community members (Delmas *et al*. 2019) and (iii) positive (symbiosis) and negative (e.g. pathogenicity) interactions between community members (Röttjers and Faust 2018).

This study investigates depth dependent associations between microbial taxa bacteria, fungi and non-fungal micro-eukaryotes (referred to in the following as ‘micro-eukaryotes’) in boreal soil and their inter-kingdom co-occurrences patterns. Previous studies have largely focused on a specific organismal group, such as fungi, but here we wanted to assess all the major microbial groups together. This is done by DNA metabarcoding analyses of bacteria, fungi and micro-eukaryotes from various depths in forest soils of five native deciduous birch (*Betula pubescens* Ehrh.) forests in Western Norway. Our study was aimed 1) to investigate how the various organismal groups differ in composition and diversity through the forest soil column down to 30 cm mineral soil depth and what are their drivers? We here expect that all groups respond strongly to soil depth due to variation in soil nutrients and pH, with highest richness in the top layer; and 2) to assess how inter-kingdom co-occurrence patterns and network architectures, indicative of biotic interactions, vary with soil depth. We expect that the species are more filtered by stressful abiotic conditions and have less biotic interactions in the deep soil profiles, while biotic interactions are more intense in the top layers.

## MATERIALS AND METHODS

### Site description, experimental setup and sampling

This study was conducted in native deciduous birch (*Betula pubescens* Ehrh.) forests at five locations in western Norway (61°30' N, 6°12' E; Material and methods S1; Fig. S1, Supporting Information). Distance between the northernmost (Molde) and the southernmost (Jøster II) locations is approx. 320 km. All the locations are positioned in the middle boreal vegetation zone, and occasional grazing as well as selective cutting have occurred through time. At all locations the bedrock is covered by thick moraine deposits (NGU 2020). The soil texture is sandy loam at all locations except for Stranda, where, in addition to the sandy loam, parts of the soil profile are dominated by silt loam. Generally, the soil chemistry did not differ significantly between locations except C: N ratio (Kjønaas et al, submitted). The ground vegetation was dominated by a mix of bilberry (*Vaccinium myrtillus* L.), grasses, herbs and bryophytes.

At each location, three 144 m^2^ plots were established within a birch stand in areas with relatively homogeneous soil, topography and vegetation. In July 2016, 20 soil cores per plot were collected down to approx. 30 cm soil depth in a grid sampling design by use of a cylindrical auger (Ø = 2.6 cm). Each soil core was divided into four layers: the forest floor (LFH) and three mineral soil layers based on sampling depth (0-5 cm ‘M1’; 5–15 cm ‘M2’; 15–30 cm ‘M3’). M1 is the organic-mineral interface layer, also termed the ‘Ah’ layer. Samples from each plot and layer were pooled into one composite sample, resulting in altogether 60 samples (5 locations*3 plots*4 depths). After pooling of subsamples and thorough homogenization, the mineral soil samples from each layer were divided into two separate samples (one for DNA/ergosterol and other for chemical analyses). As the LFH sample was difficult to homogenize, the entire sample was allocated to DNA and ergosterol analyses. For chemical analyses of the LFH layer, an additional sample was collected adjacent to the sample collected for DNA analyses using a cylindrical auger (Ø = 6.6 cm). All the samples for DNA and ergosterol analyses were stored at −20°C immediately after collection, whereas the samples for soil chemistry were kept cool during transport and frozen after returning to the lab.

Prior to the DNA and ergosterol analyses, the soil was homogenized by sieving (2 mm sterilised sieve), followed by freeze-drying and pulverizing using FastPrep instrument (MP Biomedicals, Illkirch-Graffenstaden, France). The finely grounded soil fractions were used for analysis of DNA as well as total ergosterol (fungal biomass proxy) using the protocol of Ransedokken *et al*. (2019).

### Soil chemistry analysis

Prior to analyses, the soil samples were thawed, air dried and sieved through a 2 mm sieve. The fine soil fraction was analysed for dry matter (105°C),  total C% and N% (Elementar Vario EL with TCD detector), pH (H_2_O) (PHM 220) and exchangeable elements (Hydrogen (H), Calcium (Ca), Potassium (K), Magnesium (Mg), Manganese (Mn), Sodium (Na), Phosphorous (P) and Sulphur (S)) (in 1M NH_4_NO_3_; Thermo Jarell Ash ICP-IRIS HR Duo). The fine soil fraction was finely ground (planet mill) before the C and N analysis. For details see Ogner *et al*. (1999).

### DNA extraction and Illumina sequencing

One gram of homogenized soil was added in 10 ml CTAB buffer, and 600 μl of the CTAB/soil-sludge was transferred to a 2 ml eppendorf tube containing two tungsten-carbide beads. Samples were grinded for one minute (25 Hz) and repeated after flipping the racks, and immediately stored at −20°C. DNA was extracted following the CTAB/chloroform extraction protocol and further purified using the E.Z.N.A soil DNA kit (Omega Biotek, USA) following the manufacturer's protocol. Detailed information about PCR settings and reaction is provided in supplementary material (Material and methods S1). Molecular data was generated from three full Flow-Cell runs and Paired-End (PE: 2 × 300 bp) sequencing with Illumina Miseq. Different primer combinations were used to amplify the 16S rRNA gene for bacteria ITS2 gene for fungi, and 18S rRNA gene for both fungal and micro-eukaryotes. The ITS region, which is the most common DNA barcode for fungi (Schoch *et al*. 2012), is mostly used to analyze fungal communities (Nilsson *et al*. 2009). However, various biases, including primer mismatches and length differences, may lead to some groups being excluded (Bellemain *et al*. 2010; Schadt and Rosling 2015; Tedersoo and Lindahl 2016). Universal 18S primers were used to amplify the overall micro-eukaryotic communities, also including fungi (Material and methods S1). While the highly variable ITS2 marker provide detailed taxonomic information about fungi, mainly at species and genus level, 18S provides taxonomic assignments at higher taxonomic levels. However, the more conserved 18S marker provides a more comprehensive overall picture for micro-eukaryotes, because of less amplification biases (Hugerth *et al*. 2014). The fastq-formatted sequence data sets for 16S, ITS and 18S markers gene along with barcode mapping files and associated metadata were archived at Zenodo (https://zenedo.org), a scientific data repository developed by CERN, with a single DOI for the project (10.5281/zenodo.4 415 050).

Downstream analyses focused on all the three main microbial groups bacteria, fungi and the highly polyphyletic micro-eukaryotes. The 18S data set contained both fungal and non-fungal reads therefore it was divided into a fungal and a non-fungal data set (the latter hereafter referred to as micro-eukaryotes, for simplicity).

### Bioinformatics analyses

To enable comparisons across all data sets, the same bioinformatics pipeline was employed for all data. Raw data was passed through *BayesHammer*, a bayesian clustering based error correction method (Nikolenko, Korobeynikov and Alekseyev 2013), before merging the PE reads using *PEAR* v0.9.10 with minimum overlap of 10 bp and Q20 quality score threshold for trimming the low quality part of a read (Zhang *et al*. 2014). For excluding reads with poor quality we used *FASTX*-*Toolkit* v0.0.14 (*fastq_quality_filter*, http://hannonlab.cshl.edu/fastx_toolkit/index.html) with the parameter settings: minimum Phred quality score = 30, and proportion of bases that must have minimum quality score = 0.9. A second level of quality control was performed using *VSEARCH* v2.4.3 (Rognes *et al*. 2016) to remove reads with ambiguous base = 0, length <100 bp and total expected errors (E) >0.5 for all bases. Remaining high quality reads were demultiplexed using the *SDM* v1.41 program embedded in the LotuS pipeline (Hildebrand *et al*. 2014). Using *FQGREP* v0.4.4 (https://github.com/indraniel/fqgrep) and *FASTX*-*Toolkit*, reads were oriented in the same direction and primers were trimmed. For fungal ITS data set, the ITS2 region was extracted using *ITSx* v1.0.11 (Nilsson *et al*. 2010), followed by removal of reads <100 bp. We then used *VSEARCH* for dereplication, global singletons removal and clustering (97% similarity threshold for the 16S and ITS2 data sets and 98% for 18S data set). The most abundant sequence of each cluster was designated as the representative sequence. Chimera checking was performed on the representative sequences using *uchime_denovo* algorithm (Edgar *et al*. 2011), implemented in *VSEARCH*, with the minimum divergence parameter = 0.8, abundance skew = 2 and minimum difference in segment = 3. Since we wanted to focus on the more abundant microorganisms, Operational Taxonomic Units (OTUs) with <10 reads were removed, also in order to minimize the impact of sequencing and PCR errors. Taxonomic assignment were made by comparing the representative sequence against the curated reference databases *GREENGENE* v13.8 (DeSantis *et al*. 2006; McDonald *et al*. 2012), *UNITE* v6 (Kõljalg *et al*. 2013) and *PR2* v4.62 (Guillou *et al*. 2013) for bacteria, fungi and micro-eukaryotes, respectively. In order to assign a functional guild, the fungal ITS2 OTUs were passed through *FUNGUILD* (Nguyen *et al*. 2015).

Bacteria, fungi (18S) and micro-eukaryotes (Fig. S2a, e and g, Supporting Information) showed non-normal distribution of reads, which was not the case for the ITS2 fungal data set (Fig. S2c, Supporting Information). The distribution patterns of reads per OTUs was skewed with few dominating and a long tail of rare OTUs for all groups (Fig. S2b, d, f and h, Supporting Information). Sample-based rarefaction curves of OTU richness showed that the complete richness was not captured in most samples (Fig. S3a–d, Supporting Information). Correspondingly, there was a positive and significant relationship between OTU richness and sequencing depth for bacteria (R^2^ = 0.58; *P* < 0.001), fungi (18S) (R^2^ = 0.52; *P* < 0.001) and micro-eukaryotes (R^2^ = 0.74; *P* < 0.001). Probably due to better sequencing depth, this was not observed in the fungal ITS2 based data set (R^2^ = 0.16; *P* = 0.328). To correct for potential sequencing depth biases, all data sets were rarefied prior to diversity analysis (reads per sample for bacteria, fungi (ITS), fungi (18S) and micro-eukaryotes were 6343 (16S), 55 485 (fungi ITS2), 2783 (fungi 18S) and 3430 (micro-eukaryotes), respectively. Two samples with low read numbers were discarded.

### Statistical analyses

Unless stated otherwise, statistical analyses were performed in *R* v3.5.0 (R Core Development Team 2018). Prior to analyses, all soil variables were logarithm-transformed and a Principal Component Analysis (PCA) was used to assess tentative collinearity between environmental variables and soil depth. OTU table containing species data were arcsine-transformed prior to analyses to improve variance homogeneity.

ANOVA followed by Tukey's HSD post-hoc test (package *agricolae* (Mendiburu and Simon 2015)) was used to examine differences in soil properties, ergosterol content, as well as richness, Shannon diversity and evenness of all the microbial groups, with soil depth. The same test, with Benjamini–Hochberg FDR correction, was used to assess whether the relative proportion of different phyla (from all data sets) and genera varied with soil depth. The results were illustrated using bar plots (phyla) and hierarchical heat plots (genera).

The Bray–Curtis dissimilarity index was used to generate community distance matrices. To address the relative importance of the soil chemistry variability index (i.e. PC1), and the location effect (i.e. PC2) on community composition of all groups of microorganisms, we used multivariate permutational analysis of variance (PERMANOVA), as implemented in the Adonis function of the package *vegan* (Oksanen *et al*. 2013). PERMANOVA analyses with 9999 permutations were performed using a forward selection procedure to optimise the final model (Blanchet, Legendre and Borcard 2008). We first tested model for individual above mentioned variables and included significant variables in the final model in order of their R^2^ values, to assess if remaining variation can also be explained by other variables. Nonmetric Multidimensional Scaling (NMDS) analyses were used to visualize the relative effects of these variables on microbial communities using the metaMDS function of the package *vegan*. Vectors and centroids of the variables were fitted into NMDS plots using the function envfit, and the ordiellipse function was used to plot the 95% confidence intervals (CI) of the depth.

### Network analyses

To investigate inter-kingdom co-occurrences patterns, network analyses were performed on core communities (OTUs with >0.5% of total reads, and present in at least three samples) selected from each normalised data set (Material and methods S1). These core OTUs from the three data sets were summarized at the genus level and the samples were normalized separately by subsampling to the lowest number of sequences across all three data sets. The subsampled genus-tables were then merged into one table containing genera of 93 bacteria, 73 fungi and 10 micro-eukaryotes. Co-occurrence networks for the overall data set, as well as for the four soil depths separately, were constructed with *SparCC*, as implemented in the R package *SpiecEasi* (Kurtz *et al*. 2015). *SparCC* was run with default settings and 500 bootstraps. Only associations with pseudo *P*-value < 0.05 and correlations > |0.7| were kept. Visualization of networks as well as calculation of network statistics were done with *Cytoscape* v3.6.1 (Smoot *et al*. 2011) and the R package *igraph* (Yu, Chen and Guo 2009). We calculated the flowing network characteristics: density, degree, neighbourhood connectivity, clustering coefficient and average path length (Material and methods S1). *Network density* is calculated as the proportion of realised possible correlations given the number of genera in the network; *degree* is a measure of how many correlations each genus form with other genera; *neighbourhood connectivity* measures how many correlations a ‘neighbour’ genus (i.e. one that is correlated to the focal genus) in turn is correlated to; *clustering coefficient* describes whether the network can be sectioned into groups of highly correlated organisms; while *average path length* is the distance (counted as number of edges) between all pairs of associated genera (nodes) divided by the number of genera in the network. A Wilcoxon signed-rank test was used to test whether the network characteristics differed between soil depths.

## RESULTS

### Data characteristics

**Figure 1.**
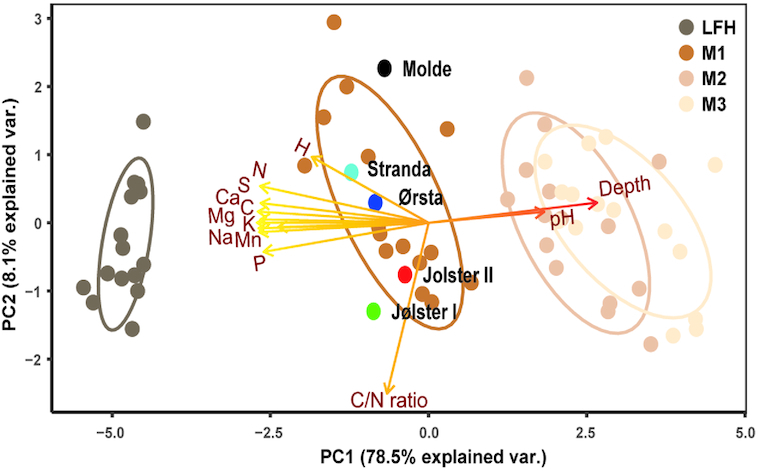
Principal component analysis (PCA) of soil chemistry data along depth gradient. Samples were collected from five different location (Molde, Stranda, Ørsta, Jølster I and Jølster II), along forest floor (LFH) and three mineral soil layers: 0–5 cm (M1), 5–15 cm (M2), 15–30 cm (M3)). The first PC axis (PC1) score of each plot (shown with dark brown to light brown colored circles) was used as a ‘*soil chemistry variability index*’, as it reflects variation related to the soil nutrients. The second PC axis (PC2) score of each plot was considered as ‘*location effect*’ (it reflects variation in C: N ratio, related to sampling locations). Ellipses indicate 95% confidence intervals around centroids for each soil depth and black, skyblue, darkblue, red and green colored circles indicate centroid for each sampling location. Following soil chemistry data was used in the analysis: total carbon (C%), total nitrogen (N%), C/N ratio, soil pH and exchangeable elements H, P, Mn, Ca, Mg, Na, S and K.

Diverse prokaryotic and eukaryotic communities, with altogether 1540 bacterial, 4388 fungal (3461 ITS-based; 927 18S-based) and 2025 micro-eukaryotic OTUs, were detected in the 60 composite soil samples (see Results S2 for details). Ergosterol content, a proxy for fungal biomass, was significantly higher in the upper litter and humus (LFH) layer (0.158 mg g^−1^) compared to the mineral soil layers (<0.026 mg g^−1^; Fig. S4, Supporting Information). Likewise, the total C and N content, as well as all exchangeable elements were highest in the LFH layer and decreased with soil depth, while pH had an opposite trend (Fig. S5, Supporting Information). PCA clearly showed that soil depth, as well as all measured edaphic factors, including pH, correlated tightly with the first PC axis (Fig. 1), which can be interpreted as a ‘*soil chemistry variability index*’. The second PC axis reflected site specific effects, including variation in the C: N ratio across plots and locations. Due to the high collinearity between numerous variables as well as soil depth, PC axes 1 (‘*soil chemistry variability index*’) and 2 (‘*location effects*’) were used in the further analyses as proxies for environmental variability.

### Microbial diversity and communities’ patterns with soil depth

**Figure 2.**
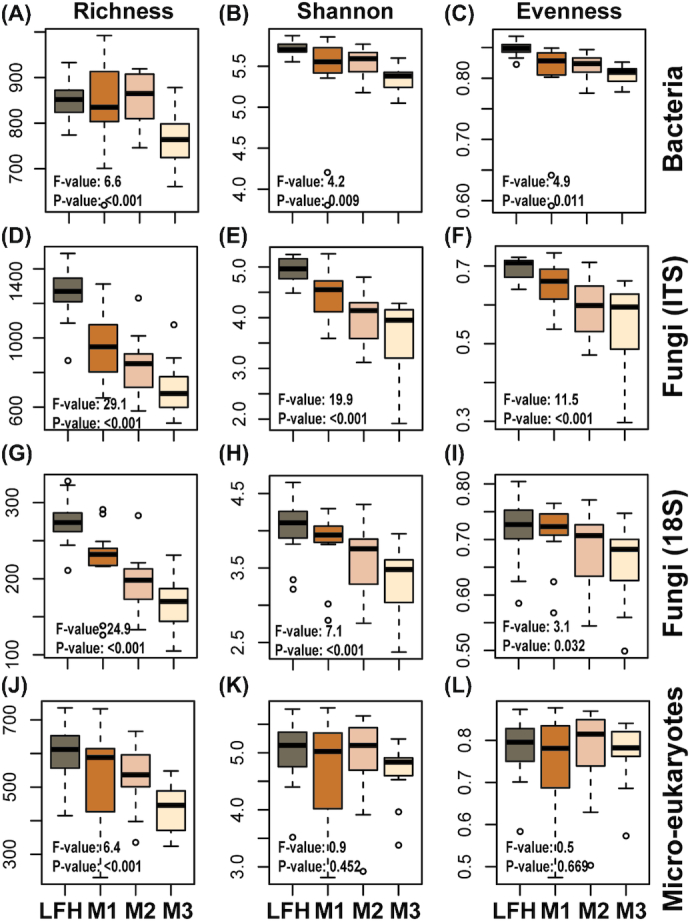
Boxplots showing soil depth related diversity patterns for different microbial groups. Diversity measure such as richness, Shannon index and evenness are shown for bacteria (16S; **A–C**), fungi (ITS: d-f; 18S: **G–I**), and micro-eukaryotes (18S: **K–L**) are shown. Samples were collected along a soil depth gradient (forest floor (LFH) and three mineral soil layers: 0–5 cm (M1), 5–15 cm (M2), 15–30 cm (M3)) from natural birch forest. Statistically significant differences among soil depth were analysed using ANOVA and Tukey's post-hoc test.

In line with depth driven changes in soil chemistry, we observed a decrease in richness with soil depth for all microbial groups (Fig. 2A, D, G and J). A similar declining trend was observed for Shannon diversity and the evenness index for all microbial taxa, except for the micro-eukaryotes (18S). However, a positive relationship (R^2^ = 0.11; *P*-value < 0.001) between micro-eukaryotic reads and ergosterol content was observed.

**Figure 3.**
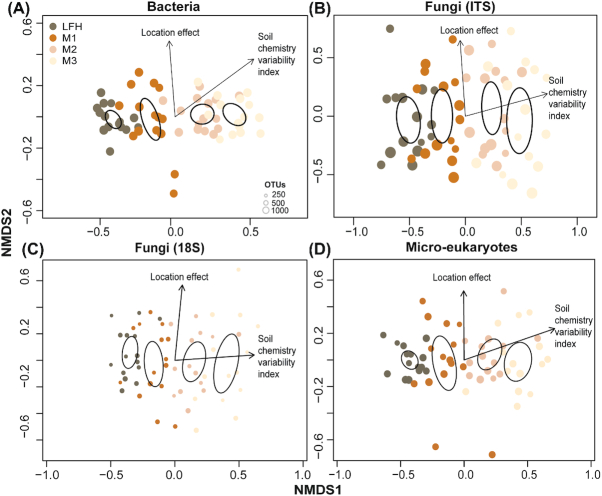
Nonmetric multidimensional scaling (NMDS) plots displaying the community structure of the different microbial groups. Bacterial **(A)**, fungal (ITS: **B**; 18S: **C**) and micro-eukaryotic **(D)** community compositional patterns among samples from different soil depth (forest floor (LFH) and three mineral soil layers: 0–5 cm (M1), 5–15 cm (M2), and 15–30 cm (M3)), as revealed by NMDS ordination analysis. The ordination plots are based on all Operational Taxonomic Units (OTUs) present in the respective microbial groups. The stress value for NMDS ordination was 0.104 for bacteria, 0.166 for fungi (ITS), 0.193 for fungi (18S) and 0.135 for micro-eukaryotes. Ellipses indicate 95% confidence intervals around centroids for each soil depth. Arrows point in the direction of maximum increase of the variables and size of circle indicates number of OTUs richness. All variables and factor shown in the panels had significant effects (*P* < 0.05) on the ordination configuration.

All the studied microbial groups showed a strong and consistent pattern of compositional shift with the soil chemistry variability index, as revealed by multivariate PERMANOVA (Table 1) and visualised using NMDS analyses (Fig. 3A–D). The location effect, linked to variability in C: N ratio among sites, had weaker relationships with the community composition. The proportion of OTUs shared among all four soil depths were higher for bacteria (86%; Fig. S6a, Supporting Information) compared to fungi (ITS: 56%; 18S: 48%; Fig. S6b and c, Supporting Information) and micro-eukaryotes (50%; Fig. S6d, Supporting Information).

**Table 1.** Results from PERMANOVA analyses, testing to which degree soil chemistry variability index (PC axis 1) and location effect (PC axis 2) can explain compositional variation in the different microbial groups.

Variables	Df	SS	MS	F-model	R^2^	*P*-value
Bacteria						
Soil chemistry variability index	1	1.76	1.76	33.22	0.35	**<0.001**
Location effect	1	0.31	0.31	5.84	0.06	**<0.001**
Residuals	57	3.03	0.05	0.59		
Total	59	5.10	1			
Fungi (ITS)						
Soil chemistry variability index	1	2.13	2.13	11.92	0.15	**<0.001**
Location effect	1	1.60	1.60	9.00	0.12	**<0.001**
Residuals	57	10.17	0.18	0.73		
Total	59	13.90	1.00			
Fungi (18S)						
Soil chemistry variability index	1	1.42	1.42	10.23	0.14	**<0.001**
Location effect	1	0.92	0.92	6.58	0.09	**<0.001**
Residuals	55	7.66	0.14	0.77		
Total	57	10.00	1			
Micro-eukaryotes						
Soil chemistry variability index	1	1.84	1.84	13.13	0.18	**<0.001**
Location effect	1	0.56	0.56	3.98	0.06	**<0.001**
Residuals	55	7.71	0.14	0.76		
Total	57	10.11	1.00			

**Figure 4.**
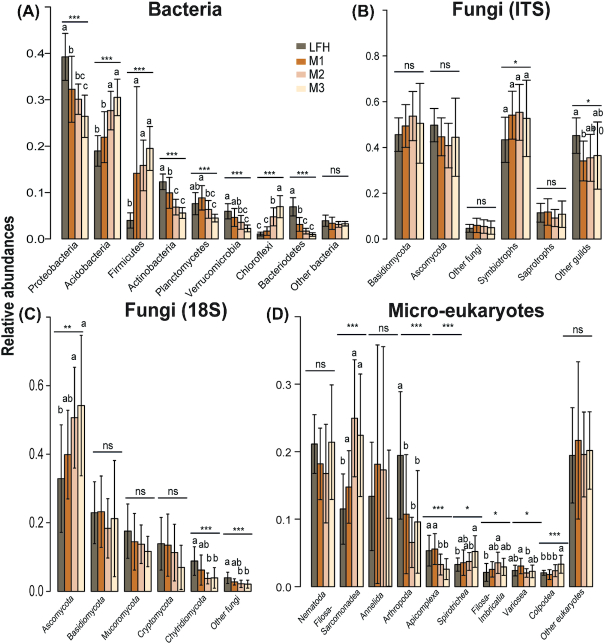
Barplots displaying abundances distribution of the different microbial taxa (phyla). Plots for relative abundances of the bacterial **(A)**, fungal (ITS: **B**; 18S: **C**), and micro-eukaryotic **(D)** taxa with soil depth (forest floor (LFH) and three mineral soil layers: 0–5 cm (M1), 5–15 cm (M2), and 15–30 cm (M3)) are shown here. Statistically significant differences among soil depth was analysed using ANOVA and Tukey´s post-hoc test. Note the significant difference in distribution of fungal groups (Ascomycota) as revealed by the 18S rRNA gene markers (C) but pattern absent while using ITS (B).

**Figure 5.**
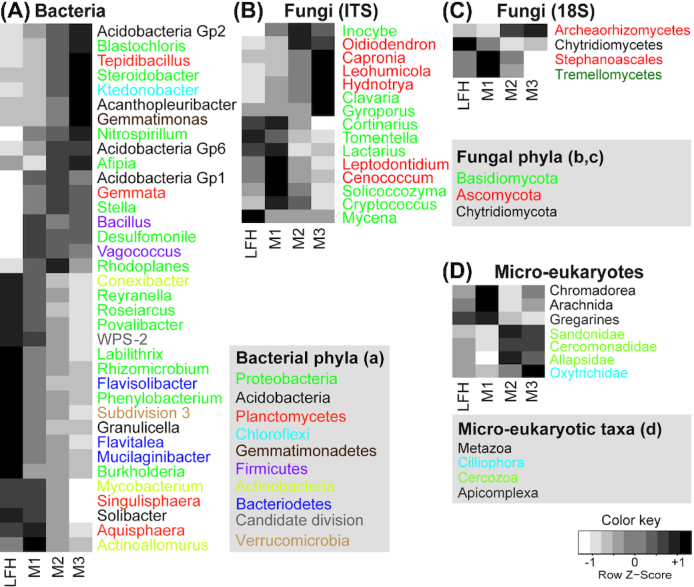
Hierarchical heatplots illustrating abundances distribution of the different microbial taxa (genus or class level). Plots for bacterial **(A)**, fungal (ITS: **B**; 18S: **C**) and micro-eukaryotic **(D)** taxa with different soil depth (forest floor (LFH) and three mineral soil layers: 0–5 cm (M1), 5–15 cm (M2), and 15–30 cm (M3)) are shown here. All taxa shown in the plots are analysed using ANOVA and Tukey´s post-hoc test and differ significantly (*P* <<0.05i>) in abundances among soil depth. Colour gradients in the plots from white to grey to black indicates increasing dominance of the taxonomic groups in particular soil depth. Different color in bacterial and fungal genus name indicates respective phyla and in case of micro-eukaryotes color represent different taxa at higher taxonomic level. Different colors are used to indicate phyla for bacteria, fungi and micro-eukaryotes

Overall, the prokaryotic communities were dominated by Proteobacteria (32% reads; 31% OTUs) and Acidobacteria (25% reads; 21% OTUs). The shift in bacterial composition from organic to mineral soil layers was mainly driven by changes in Proteobacteria, Actinobacteria, Verrucomicrobia and Bacteriodetes, being significantly more proportionally abundant in the upper LFH layer, while Acidobacteria, Firmicutes and Chlorofexi were proportionally more abundant in the deeper mineral soil layers (Fig. 4A). Similarly, proportions of certain genera also showed consistent patterns with soil depth (Fig. 5A). The proportions of the genera *Povalibacter*, *Roseiarcus, Rhizomicrobium*, *Reyranella*, *Burkholderia* and *Phenylobacterium* (Proteobacteria); *Granulicelia* (Acidobacter) were significantly higher in upper LFH layer (Fig. 5A) while the proportions of *Steroidobacter*, *Nitrospirillum, Blastochloris* and *Afipia* (Proteobacteria); *Gp2*, *Gp6* and *Acanthopleuribacter* (Acidobacteria) and *Tepidibacillus* (Planctomycetes) were higher in the lower mineral layers.

As revealed by the 18S primers, fungi dominated among the eukaryotes, making up 50% of the reads and 30% of the OTUs. In this data set, the proportion of Ascomycota reads was significantly higher in the lower mineral soil layer compared to the upper LFH layer (Fig. 4C), a shift largely driven by significantly higher proportions of Archaeorhizomycetes reads in the deeper mineral soils (Fig. 5C). In contrast, Chytridiomycota (Fig. 4C) made up a significantly higher proportion of reads in the upper organic layer, while the other fungal phyla, Basidiomycota, Mucoromycota and Cryptomycota, showed rather uniform distribution with soil depth in the 18S data set. When the ITS2 region was used to survey the fungal communities, a contrasting pattern was detected for some groups (Figs 4B and 5B), especially so for the Archaeorhizomycetes, which was only recovered as dominating taxa while using the 18S primers (Fig. 5C). In contrast to the 18S data, basidiomycetes (50% reads; 29% OTUs) were the most abundant in the ITS2 data, but ascomycetes still included a higher OTU richness (45% reads; 60% OTUs). In the ITS2 data set, relatively higher proportion of symbiotrophic fungi such as *Inocybe*, *Gyroporus* (both ECM) appeared in the mineral soil layers (Figs 4B and 5B). Several fungal genera had significant shifts in proportions with soil depth (Fig. 5B). *Clavaria* were proportionally more abundant in the lower mineral layer, whereas *Cortinarius*, *Lactarius, Tomentella* (all ECM) and *Mycena* were proportionally more abundant in upper LFH layer. The Ascomycota genera *Oidiodendron*, *Capronia*, *Leohumicola* and *Hydnotrya* (ECM) were proportionally more common in the lower mineral layer.

Among the other micro-eukaryotes, Metazoa was the most dominating group (28% reads; 17% OTUs) followed by Cercozoa (11% reads; 27% OTUs) and Ciliophora (4% reads; 8% OTUs). As for bacteria and fungi, they all displayed significant depth related changes in their proportions (Fig. 4D). In the upper layer, the proportion of metazoan reads, and specifically so Arachnida, was significantly higher along with Conosea (Variosea). Additionally, apixomplexan parasites belonging to Gregarines were also proportionally more abundant in the upper organic layer. The cercozoans Filosa-sarcomonadea (Sandonidae, Cercomonadidae and Allapsidae) and Filosa-Inbricatia, and the ciliates Spirotrichea (Oxytrichidae) and Colpodea made up a higher proportion of the reads in the lower mineral layers (Fig. 5D).

### Inter-kingdom co-occurrence patterns with soil depth

**Figure 6.**
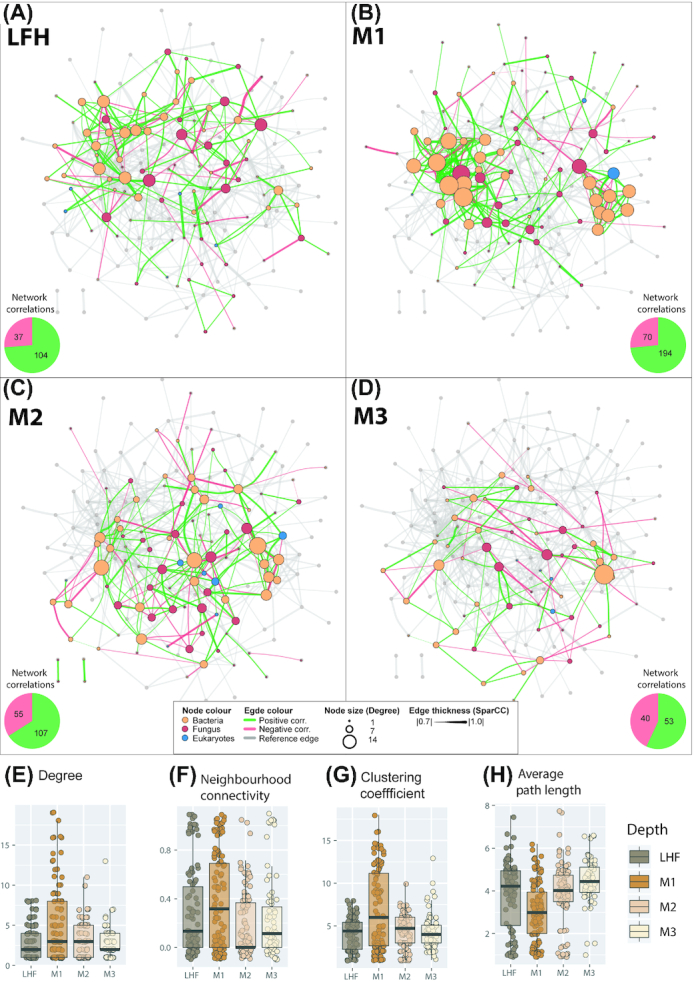
Inter-kingdom correlation patterns of bacterial, fungal and eukaryotic genera. The network is based on a SparCC correlation analysis for the forest floor (LFH) **(A)** and mineral soil layers 0–5 cm (M1) **(B)**, 5–15 cm (M2) **(C)** and 15–30 cm (M3) **(D)**. Pie charts from each panel represents number of total correlations (positive as green and negative as red) in Network from each depth. Positive correlations (SparCC > 0.7, *P* < 0.05) are drawn as green edges and negative correlations (SparCC < 0.7, *P* < 0.05) are drawn as red edges. Nodes represents genera and are coloured according to taxonomic group: bacteria in orange, fungi in red and micro-eukaryotes in blue. The size of a node is proportional to connection it forms with other nodes. The thickness of the connection between two nodes is proportional to the value of correlation coefficients. The network in transparent grey is a reference network combining the correlations for all four depth layers. Boxplots **(F–H)** show the upper and lower quartile, and the average value for important network statistics for all four depths: degree **(E)**, neighbourhood connectivity (F), clustering coefficient (G) and average path length (H). The *degree* of a genus is the number of co-occurrences it has with other genera (i.e. the number of connections (edges) formed by a node to other nodes). The *neighbourhood connectivity* is the average connectivity (correlations) of neighbours of a given node (i.e. nodes correlated to a given node can themselves be correlated to other nodes). The *clustering coefficient* describes whether the network can be sectioned into clusters of highly interconnected organisms. Highly clustered networks are those that contain groups of statistically associated organisms. A high clustering coefficient is an indication of a high degree of interactions and associations. The *Average path length* is the distance (counted as number of edges) between all pairs of associated genera (divided by the number of genera in the network. A low average path length indicates that most species in the network are connected through a few intermediates’ species.

The network analyses revealed a higher number of positively or negatively correlated (co-occurring) genera in the top layers, compared to the deepest layer M3, with M1 having the highest number of correlations (Fig. 6A–D, Figs S7–S10, Supporting Information). In line with this, the network statistics *network density, degree* and *neighbourhood connectivity* were on average highest in M1 (Table S1, Supporting Information; Fig 6E and F). Likewise, the average *path length* was lowest in the M1 layer and increased with increasing depth (Fig. 6h). A low average *path length* indicates that most taxa in the network are connected through few intermediate taxa. The network statistics suggest that organisms in the M1 layer form more association with each other compared to in the other soil layers. Further, the clustering coefficient, which describes whether the network can be sectioned into clusters of highly correlated genera, was also highest in M1 (Fig. 6G). A Wilcoxon signed-rank test showed that the M1 layer was significantly different for most of the network statistics (Table S2, Supporting Information) and 14 genera were exclusively detected in this layer (Fig. S11, Supporting Information). There was no significant difference between the LFH layer and the M3 layer in the network topology (Table S2, Supporting Information). These two layers have 30 genera in common (Fig. S11, Supporting Information), and only 10 and 6 genera were exclusively detected in the LFH and M3 layers, respectively. These two layers are thus predominantly dominated by similar taxonomic composition. The proportion of positive correlations was highest for the above LFH (73.8%) and M1 layers (73.5%), and decreased towards the M3 layer (57.0%), where the relative proportion of negative correlations was higher (43%) (Fig. 6A–D). Taxa belonging to Firmicutes (*Vagococcus, Bacillus, Enterococcus, Paenibacillus* and *Macrococcus*), Acidobacteria (*Gp1* and *Gp3*) and a yeast group (*Candida*), showed higher numbers of co-occurrences in the upper M1 compared to other layers.

## DISCUSSION

As hypothesised, soil depth represents a strong and complex environmental gradient with multiple edaphic factors changing concurrently with depth in the investigated birch stands. A strong niche partitioning with depth is reflected in both diversity and structure of the bacterial, fungal as well as micro-eukaryotic communities. Further, the soil gradient also affected inter-kingdom co-occurrence patterns among soil horizons, and most dense as well as complex network, with higher degree and neighborhood connectivity, was observed in the upper mineral soil layer (M1).

In addition to soil depth, some of the variation in community composition could be attributed to environmental differences among locations (location effects), including differences in C: N-ratio, aspect and latitude. Two locations (Molde and Stranda) were south-facing, two (Jølster I and II) north-facing and the last one (Ørsta) east-facing, which lead to temperature differences. The systematic variation in C: N-ratio may be induced by a gradient in N deposition (Fischer *et al*. 2007).

The two different markers (ITS2 vs 18S) used for fungi provided a slightly different view of the fungal communities, especially when it comes to Archaeorhizomycetes (Ascomycota) and chytrids. These two groups were not captured fully by the ITS2 primers, indicating primer biases discriminating against e.g. Archaeorhizomycetes and chytrids (Rosling *et al*. 2011; Ihrmark *et al*. 2012; Tedersoo *et al*. 2014; Schadt and Rosling 2015; Nilsson *et al*. 2019). The 18S universal primers employed in this study seems to amplify most eukaryotes (Hadziavdic *et al*. 2014), providing a more comprehensive picture of the fungal community composition compared to the ITS2 data set. However, there might also be primer biases associated with the 18S primers (Anderson, Campbell and Prosser 2003). Nevertheless, our results highlights the importance of using different markers to better capture the diverse microbial communities in soil.

### Depth matters: biomass, diversity patterns and soil characteristics

One of the most evident changes through the soil profile was the distinct decrease in fungal biomass with depth in the deciduous forest, corroborating findings of previous studies from coniferous forest (Hartmann *et al*. 2012; Voříšková *et al*. 2014). A similar declining trend in bacterial biomass with depth in deciduous forest soils has also been reported (Hartmann *et al*. 2012). Using traditional culturing and quantitative methods, a sharp decline in protozoan biomass with depth has been observed in Danish deciduous and coniferous forest sites (Ekelund, Rønn and Christensen 2001). Thus, there is mounting evidence of a general pattern of decreasing biomass with increasing forest soil depth, regardless of microbial groups.

We observed significant declines in diversity patterns with depth across different microbial groups, with a stronger pattern for fungi compared to bacteria and other micro-eukaryotes. Our findings are in line with studies from boreal coniferous forest (Rosling *et al*. 2003; Lindahl *et al*. 2007; Baldrian *et al*. 2012; Hartmann *et al*. 2012; Santalahti *et al*. 2016; Kyaschenko *et al*. 2017). Similarly, studies from deciduous mountainous hardwood (Du *et al*. 2017), temperate oak forest (Voříšková *et al*. 2014) and tallgrass prairie (Jumpponen, Jones and Blair 2010) ecosystem also consistently revealed declining patterns for fungal richness. These patterns are not surprising considering the observed lower biomass of fungi (this study) and bacteria (see above) in deeper mineral soil. Although micro-eukaryotes are important in soil food webs, the effects of soil depth on their diversity has rarely been investigated (Potapov *et al*. 2017). A synchronous decrease in diversity of bacteria and fungi together with micro-eukaryotes suggest key role of micro-eukaryotic organisms in the food webs since they are highly dependent on bacteria and fungi as C sources (Crowther *et al*. 2013; Geisen *et al*. 2016; Seppey *et al*. 2017; Xiong *et al*. 2017).

The variation in soil chemistry affects micro-eukaryotic diversity (Tedersoo *et al*. 2015), and the observed diversity patterns showed a high correlation with the soil gradient patterns. The C concentration and soil nutrients availability are considerably higher in upper organic layers. Further, the easily decomposable labile fraction of C has been found to decrease with soil depth at the same sampling locations (Hansen *et al*. submitted). In agreement with previous studies targeting bacterial richness from coniferous forest (Eilers *et al*. 2012; Hartmann *et al*. 2012), we found a decline in bacterial richness with soil depth. There is a large variation in microclimatic conditions and diverse resources of organic matter (Schurig *et al*. 2013) at the soil surface, that may support a high diversity of ecological niches as compared to the deeper soil. Further, only few bacterial groups are well-adapted to grow under the oligotrophic conditions that characterize the deeper mineral soil. The decline in fungal biomass and diversity in deeper mineral soil may also affect the bacterial richness, as some bacterial groups feed on fungi through their ability to secrete protein and cause hyphal decay (Swain *et al*. 2017). Together, our results indicate that variation in environmental conditions with soil depth represent an ecological filter, and that many surface-dwelling organisms do not thrive in the nutrient poor environments of the deeper soil horizons. Our results also suggest that, independent of organismal groups, a considerable part of the diversity cannot be recovered when examining only the topsoil layers, since different soil horizon host diverse microbial assemblages.

### Depth matters: community compositional patterns

Our observation of a clear shift in fungal communities with soil depth, agrees with previous studies from coniferous forests (Rosling *et al*. 2003; Hartmann *et al*. 2012; Hobbie *et al*. 2014b; Santalahti *et al*. 2016). Saprotrophic fungi, including *Mycena* spp. (common litter basidiomycetes) with demonstrated peroxidase activity (Kyaschenko *et al*. 2017) and the common leaf endophytes *Phialocephala* spp., dominated in the top layer. Here, fresh litter is supplied by aboveground vegetation and the saprotrophic fungi may out-compete the ectomycorrhizal fungi (Fernandez and Kennedy 2016). In accordance with previous studies, we detected an overall higher dominance of symbiotrophic fungi in the deeper mineral soils. However, the ectomycorrhizal genera *Cortinarius*, *Tomentella* and *Lactarius*, were significantly more abundant in the organic layer. This can be linked with the ability of *Cortinarius* and *Lactarius* to infect root tips in the organic layer and also their high extrametrical mycelium production rate generating more biomass (Genney, Anderson and Alexander 2006; Anderson, Genney and Alexander 2014). Further, some species of *Cortinarius* may secrete peroxidase in order to access and decompose organically bound N present in the organic layers (Bödeker *et al*. 2014). In an experiment using litter bags with ^15^N-labelled beech leaf, Pena *et al*. (2013) showed high abundance of *Tomentella* in leaf litter and provided experimental evidence that they are key players in decomposition and N capture from decaying leaves. On the other hand, the dominance of the ectomycorrhizal genera *Inocybe*, *Gyroporus* and *Russula* increased in the deeper mineral soil, where the substrate becomes increasingly depleted and the symbiotrophic fungi likely are highly dependent on host-derived C. This has also been validated using a isotope tracer technique, conducted at the Duke free CO_2_ enrichment (FACE) experiment (Hobbie *et al*. 2014a; Hobbie *et al*. 2014b). Here they showed that ECM fungi with hydrophobic exploration types (e.g. *Cortinarius* and *Tricholoma*) preferentially utilise N from the deeper humus layer whereas fungi with hydrophilic exploration types (e.g. *Russula* and *Lactarius*) acquired N from the forest litter layer. Further, they also showed that *Lactarius* and *Russula* only incorporated fresh photosynthate as C source (litter-derived), whereas *Inocybe* and *Cortinarius* also forage on soil-derived C. This suggests that different fungal species and genera have different niche preferences due to their nutrient acquisition strategies (Lindahl *et al*. 2007). The 18S marker demonstrated a significantly higher abundance of Archaeorhizomycetes in deeper soil. Although we lack a complete understanding of the Archaeorhizomycetes ecology (Rosling *et al*. 2011), they may act as root-associated mutualists (Menkis *et al*. 2014). The high abundance of Archaeorhizomycetes in the deeper mineral soil may also indicate an adaption to nutrient limited and stressful environments conditions, as suggested in previous studies (Sterkenburg *et al*. 2015; Pinto-Figueroa *et al*. 2019).

There has been numerous studies focusing on the vertical distribution of fungi in deciduous forest soils, but the literature is more limited for bacteria (Baldrian *et al*. 2012; Lladó, López-Mondéjar and Baldrian 2017) and specifically for micro-eukaryotes (Oliverio *et al*. 2020). As expected, these organismal groups were also structured by soil depth. Vertical differences in bacterial community composition have previously been reported from sub-boreal spruce (Hartmann *et al*. 2012) and pine dominated montane forest (Eilers *et al*. 2012), where different bacterial taxa tended to show variable abundances with soil depth. We found that the most abundant group, Proteobacteria (32% of the reads), was significantly more abundant in the organic layer of these deciduous forests, which is in agreement with previous studies in coniferous forests (Baldrian *et al*. 2012; López-Mondéjar *et al*. 2015). In these studies, however, the sampling was limited to the litter and humus layers, whereas we investigated communities down to 30 cm mineral soil depth. Changes in the relative abundance of Verrucomicrobia and Bacteriodetes with depth were particularly striking, both being more abundant in the organic layer. A similar pattern has been reported previously from coniferous forests for Bacteriodetes (Eilers *et al*. 2012). Both Bacteriodetes and Proteobacteria are typically copiotrophic bacteria, found commonly in combined C- and nutrient-rich environments (Goldfarb *et al*. 2011). Observed higher dominance of Acidobacteria taxa Gp2 and Gp6 in the C- and nutrient-poor deeper mineral soil is in agreement with Fierer, Bradford and Jackson (2007), where higher abundances was observed in C-poor bulk soils compared to rhizosphere. As the soil substrate quality become poor and resistant to degradation in deeper soil, copiotrophic bacteria are replaced by oligotrophic bacteria, such as Acidobacteria, that are able to cope with more recalcitrant substrates, or Actinobacteria with a high metabolic plasticity (VanInsberghe *et al*. 2013).

Although the diversity patterns showed limited changes with soil depth for the micro-eukaryotes, there was still decline in abundance of nematodes, arthropods and Apicomplexa with soil depth. Root-derived carbon enters the soil animal food web via different pathways: animals can either feed (i) directly on living or dead roots, (ii) on bacteria living on root exudates or (iii) on fungi that acquire carbon from roots, i.e. mycorrhizae. The positive correlation between fungal biomass and micro-eukaryotic abundance may reflect the fungi's importance in driving the community patterns. Since several eukaryotic organisms, including protists (e.g. *Cercomonas* and *Lecythium*), testate (e.g. *Cryptodifflugia*) and naked (e.g. *Acanthamoeba* and *Leptomyxa*) amoebae (Dumack, Müller and Bonkowski 2016; Geisen *et al*. 2016), exclusively feed on fungi, changes in micro-eukaryotic communities with declining fungal richness and biomass is expected. High abundance of parasitic Apicomplexa (Gregarines) in the organic layer possibly reflects host dependencies (ex. Arthropoda) in the same depth (Bates *et al*. 2013; Mahé *et al*. 2017). In contrast, the higher abundances of Catenulida (Metazoa; flat-worms) and Marionina (Annelid; ringed-worms) in mineral soil may be due to their burrowing ability.

### Depth matters: inter-kingdom co-occurrence patterns

Co-occurrence and network analyses can be used to explore putative interactions among microbial communities. Positive correlations mean that genera co-occur more than by chance, which may potentially be due to mutualism, parasitism, predation or alternatively, shared niche preferences. On the other hand, negative correlations may suggest competitive exclusion, or alternatively, preference for different niches (Röttjers and Faust 2018). Despite an overall higher microbial diversity in the forest floor (LHF), the relative amounts of positive correlations between genera was similar in the organic and the upper mineral soil, whereas the total amount of correlations (positive or negative) was higher in the upper mineral layer. In addition to the highest number of co-occurrences in the upper mineral layer, the network here was most dense, with higher degree and neighbourhood connectivity. The structural properties of network statistics suggest that organisms in the upper mineral layer form more association with each other compared to upper organic and deeper mineral soil layers. The LFH horizon is likely the most unstable and disturbed environment due to fresh litter supply from aboveground vegetation, and is highly influenced by fluctuating climatic conditions such as precipitation and temperature. Compared to forest floor, in the upper mineral soil the variability in the environmental conditions is lower whereas the amount of available plant-derived C is higher compared to deeper mineral layers. Therefore, organisms with competitive trait (C strategy) is expected to be more common in this soil layer. This variability in microbial trait strategy may result in a shift in the microbial communities from organic to the upper mineral layer. While comparing network complexity among arable, grass and forest ecosystem, Creamer et al. (2016), observed a denser microbial network in relatively stable forest soils, where a developed food web is expected. Since communities in the C-poor deeper mineral soil are relatively stable due to less environmental fluctuations and microbial organisms themselves potentially represent a relatively more important carbon source for each other (Crowther *et al*. 2013; Xiong *et al*. 2017). This may provide higher connectivity among microbial communities with more biotic interactions opportunities for C exchange and their survival (Milici *et al*. 2016). We also would like to highlight that seasonal variation may affect network architecture along depth gradients (Ings *et al*. 2009), but as our data were collected at one time point, they can to be used to validate the impact of time on the network architecture.

As expected, in the mineral soil we found that the network density as well as the proportion of positive correlations decreased downwards in the soil profile and, correspondingly, negative correlations increased. These findings are corresponding with Creamer *et al*. (2016), who showed that microbial networks are relatively denser in nutrient-rich soils, compared to nutrient-poor arable soil and positive interactions are stronger in soils with lower organic matter. The relative increase in negative correlations with soil depth may, on one hand, indicate that the relevant taxa have different niches preference, and/or that competition for the same resources is increasing with depth, as suggested by Weiss *et al*. (2016). Only a few organisms with highly specialized enzymatic capabilities can decompose organic matter in the lower mineral soil where nutrient availability is low. This, in turn, means less freely accessible C to other organisms as well as potentially lower abundance of organisms as a C source through predation and parasitism. In the lower mineral soil there is less opportunity for organisms to interact due to the lower species richness of all organismal groups, which may explain the lower network complexity in the lower mineral (M2 and M3) horizons.

Based on the network analyses alone, we cannot separate the dominant process(es) behind the positive correlations, i.e. whether this is an indication of shared niche preferences in the soil or biotic interactions in the form of synergetic relationships, commensalism, parasitism or proto-cooperation. Which process(es) are structuring the networks are also expected to vary for different organisms. For instance, members of the ubiquitous soil bacterial group Firmicutes (Barberán *et al*. 2011) such as *Vagococcus*, *Bacillus*, *Enterococcus*, *Paenibacillus* and *Macrococcus*, which are copiotrophic in nature, tended to co-occur more in the upper mineral layer than expected by chance. This may be because they share a specific (and yet undefined) niche. The soil yeast fungus *Candida*, also showed a high co-occurrence with members of Firmicutes in the upper mineral layer. This could suggest a putative role of the *Candida* yeasts as a nutrient and energy source for the predatory bacteria (Botha 2011) or vice versa, and further they can be opportunists feeding on the product decomposed by other bacterial groups (Mašínová, Yurkov and Baldrian 2018).

## CONCLUSIONS

In this study, we demonstrate vertical niche partitioning for bacterial, fungal and other micro-eukaryotic communities in forest soils, mirroring concurrent changes in soil properties (H1). Dominant taxa varied across the soil profile, and diversity declined significantly with depth independent of microbial groups. These results suggest that different soil horizons should be taken into consideration when assessing microorganisms in soil. We demonstrate that inter-kingdom co-occurrence patterns vary dependent on soil layer (H2) and the most complex network occurs in upper mineral soil layer. Although the results are correlative in nature, they provide hypotheses about depth-dependent biotic interactions in forest soils, calling for more detailed experimental studies. Based on our results we also suggest to use a broader set of primers and DNA-markers in future community studies to capture a more comprehensive picture of the soil microbiota.

## Supplementary Material

fiab022_Supplemental_File
