# Supplementary material for: Soil depth matters: shift in composition and inter-kingdom co-occurrence patterns of microorganisms in forest soils
Source: FEMS Microbiol Ecol. 2021 Feb 6;97(3):fiab022. doi: 10.1093/femsec/fiab022 (PMC7948073; doi:10.1093/femsec/fiab022)
Supplement: fiab022_Supplemental_File [file fiab022_supplemental_file.docx]

**Supplementary Information:**

**Soil depth matters: Shift in composition and inter-kingdom co-occurrence patterns of microorganisms in forest soils**

Sunil Mundra^1^*, O. Janne Kjønaas^2^, Luis N. Morgado^1,3^, Anders Kristian Krabberød^1^, Yngvild Ransedokken^4^, Håvard Kauserud^1^

*Corresponding author.

e-mail- [sunilm@ibv.uio.no](mailto:sunilm@ibv.uio.no); sunilmundra@hotmail.com

The following Supplementary Information is available for this article:

**Materials and Methods S1**

# Results S2

**Tables S1-S2**

**Figures S1-S11**

**References**

**Supplementary Information**

# Materials and methods S1

***1.1 Site description***

This study was carried out at five locations in native birch (*Betula pubescens* Ehrh.) forests in western Norway (Fig. S1a). The locations Jølster I (61°30'39’’ N, 6°17'54’’E; 250 m amsl) and Jølster II (61°30'22’’N, 6°12'46’’E; 345 m amsl) are located on a relatively steep N-facing slopes; Stranda (62°16'23’’ N, 6°51'5’’ E; 430 m amsl) and Molde (62°49'36’’ N, 7°38'58’’ E; 215 m amsl) on S-SE facing slopes, whereas Ørsta (62°9'1.8’’ N, 6°12'2’’ E; 210 m amsl) is located on a relatively gentle E-NE facing slope. The height adjusted mean annual temperature (MAT) for Jølster I, Jølster II, Ørsta, Stranda and Molde locations was 4.6 °C, 4.0 °C, 5.3 °C, 3.5 °C and 4.8 °C based on modelled estimates of temperature on a 1x1km grid, whereas mean annual precipitation (MAP) was 2307, 2481, 1923, 1458 and 1830 mm, respectively, measured at the nearest meteorological station (period: 1961–90; source: www.eklima.no). Soil texture is sandy loam at four locations except Stranda where it is sandy loam/silt loam.

## 1.2 DNA extraction and Illumina sequencing

Different primer combinations were used for amplifying 16S, ITS2 and 18S rRNA gene region for bacteria (515F 5´-GTGCCAGCMGCCGCGGTAA-3` and 806R 5´-GGACTACHVHHHTWTCTAAT-3´ (Caporaso, et al. 2011)), fungi (fITS7a 5`-GTGARTCATCGARTCTTTG-3` and ITS4 5´-TCCTCCGCTTATTGATATGC-3` (Ihrmark, et al. 2012, White, et al. 1990)) and micro-eukaryote (TAReuk454FWD1 5´- CCAGCASCYGCGGTAATTCC-3` and TAReukREV3 5´- ACTTTCGTTCTTGATYRA-3` (Stoeck, et al. 2010)), respectively. To achieve demultiplexing, a 12-bp Multiplex Identification DNA-tags (MIDs) were attached at 5’ end of both forward and reverse primer whereas for fungi and micro-eukaryote MIDs of varying size (7-9 bp) were used. We used one step PCR with tagged primers. The PCR reaction settings were similar for all three microbial group. Altogether 1 μl DNA template was used for the 25 μl PCR reaction containing 14.60 μl MQ water, 2.50 μl 10x buffer, 2.50 μl MgCl_2_, 0.20 μl dNTP’s (25 mM), 1.50 μl reverse and forward primers (10μM), 1 μl BSA (20 μg/μl) and 0.20 μl AmpliTaq Gold^®^ DNA Polymerase (5U/μl). The PCR conditions used for different microbial groups were as follows: bacteria – initial denaturation 95 °C (5 min), then 35 cycles of 95 °C (30 sec), 54 °C (30 sec), and 72 °C (90 sec), elongation at 72°C for 10 min; fungi - initial denaturation 95 °C (5 min), then 32 cycles of 95 °C (30 sec), 55 °C (30 sec), and 72 °C (1 min), elongation 72°C for 7 min; and eukaryote - a cycle of 98 °C (7 min), afterward 15 cycles of 98 °C (30 sec), 53 °C (30 sec), and 72 °C (45 sec), again 15 cycles of 98 °C (30 sec), 48 °C (30 sec), and 72 °C (45 sec) and finally ending with 72°C for 10 min cycle. Agencourt AMPure beads XP (Beckman Coulter, CA, USA) were used for purification of PCR amplicons and concentration was measured using Qubit 2.0 Flurometer (Life Technology, Carlsbad, CA, USA) to make an equimolar mix for each of the microbial groups.

## 1.3 Statistical analysis

***Core community selection and network statistic calculations***

Core community from each microbial group (bacteria, fungi (ITS and 18S) and micro-eukaryotes) was selected by removing OTUs with <0.5 % of total reads, and if not present in at least three samples. Furthermore, fungal ITS and 18S datasets were checked for duplicate entry for taxa. From fungi (18S) data, OTUs matching to Agaricomycetes, Sacharomycetes, Mucoromycetes were removed due to their better representation in fungi (ITS) data, and Archaeorhizomycetes, Chtridiomycetes, Cryptomycetes were kept due to better capture in 18S data. One Archaeorhizomycetes OTU from ITS dataset was also removed. Core OTUs from the all the microbial groups were summed at the genus level and the samples were normalized separately by subsampling to the lowest number of sequences across all the datasets. The subsampled genus-tables were then merged into one table containing 93 bacteria, 73 fungus, and 10 eukaryotic genera.

Correlation networks were constructed for each depth layer separately based on the table of genera and important network statistics were calculated for each network: *Density* is a quantification of how highly connected a network is given its size. It is the probability that two genera (represented by nodes in the network) are statistically associated through direct co-occurrences or some intermediates. A higher density for the association network implies more interactions and association per genus. The *degree* of a genus is the number of correlations it forms with other genera. I.e. it is the number of connections (edges) formed by a node to other nodes. The *clustering coefficient* describes whether the network can be sectioned into groups of highly correlated organisms. A high clustering coefficient is an indication of a high number of interactions and associations in the community. The *neighbourhood connectivity* is the average connectivity (correlations) of neighbours of a given node. I.e. nodes correlated to a given node can themselves be correlated to other nodes. A high neighbourhood connectivity is a measure of several correlations between several genera, rather than one gnus being correlated to many genera. *Average path length* is the distance (counted as number of edges) between all pairs of associated genera (nodes) divided by the number of genera in the network. A low average path length indicates that most genera in the network are connected through a few intermediates.

# Results S2

## 2.1 Sequence data and overall community composition

In the bacterial dataset, 1 220 636 reads remained after quality filtering, and clustered into 1603 OTUs. OTUs with no blast hits (36 OTUs), similarity to Archaea (24 OTUs), identity and coverage < 70% (2 OTUs) and detected in extraction control (1) were removed and 1540 OTUs (1 151 972 reads) were retained in raw abundance dataset. Average reads per samples was 19 200 (range 6343:43 666) and per OTU was 748 (range 10:56 325). Of the total 26 identified phyla of bacteria, Proteobacteria (32% reads; 31% OTUs) was the most common, followed by Acidobacteria (25% reads; 21% OTUs), Firmicutes (13% reads; 5% OTUs), Actinobacteria (9% reads; 9% OTUs) and Planctomycetes (7% reads; 11% OTUs). The 20 most abundant OTUs represented 30% of total reads.

The whole 18S dataset contained 4 398 576 reads after quality filtering, and clustered into 3412 OTUs. OTUs with no blast hits and plant origin (176), identity and coverage < 70% (284) were removed; and 2952 OTUs (2 448 948 reads) were retained in whole 18S dataset. Of the total reads across the whole 18S dataset, 50% (31% OTUs) matched to fungi, therefore 18S dataset was divided into two different taxonomic group “micro-eukaryotes” and “fungi (18S)”.

Micro-eukaryotes contained average reads per samples 19 994 (range 58:89 174) and per OTU was 592 (range 9:165 173). Metazoa (53% reads; 25% OTUs) was the most dominating groups among micro-eukaryotes followed by Cercozoa (24% reads; 38% OTUs), Cilliophora (9% reads; 11% OTUs), Apicomplexa (4% reads; 4% OTUs), Conosa (2% reads; 5% OTUs) and Ochrophyta (2% reads; 3% OTUs). OTUs from additional 20 other micro-eukaryotic groups were detected with an abundance < 1% reads. Among the 20 most abundant OTUs, 15 belonged to Metazoa; each of Cercozoa and Cilliophora comprised two OTUs and one OTU belonged to Apicomplexa. They accounted for 35% of total reads.

Fungi (18S) contained average reads per samples 20 822 (range 74:59 937) and per OTU was 1347 (range 12:126 417). Of the total 9 identified phyla of fungi, ascomycetes (44% reads; 20% OTUs) were the most abundant, whereas OTUs count was more in case of basidiomycetes (21% reads; 24% OTUs), followed by zygomycetes (14% reads; 11% OTUs), cryptomycetes (11% reads; 17% OTUs) and Chytridiomycota (6% reads; 16% OTUs). Glomeromycota, Zoopagomycota, Kickxellomycota and Entomophthoromycota were also detected in very low proportion (<1% of the total reads). Among the 20 most abundant OTUs, nine belonged to *Archaeorhizomyces*, two beloged to *Russula* and two belonged to *Mortierella* genera. They accounted for 56% of total reads.

In case of fungal (ITS) data 8 566 734 reads remained after quality filtering, and clustered into 4825 OTUs. OTUs with no blast hits and similarity to plants and protist (1197), identity and coverage < 70% (166) were removed; and 3461 OTUs (6 398 343 reads) were retained in raw abundance dataset. Average reads per samples was 106 639 (range 55 485:167 791) and per OTU was 1849 (range 1:280 288). Of the total 6 identified phyla of fungi, basidiomycetes (50% reads; 29% OTUs) were the most abundant, whereas ascomycetes were more in numbers as reflected by the higher number of OTUs (45% reads; 60% OTUs). Low abundance was observed for Mucoromycota (2% reads; 4% OTUs) as well as Rozellamycota, Glomeromycota and Chytridiomycota (< 1% reads). Unclassified fungi covered 3% of total reads as well as OTUs. The 31% of the total reads represented the 20 most abundant OTUs.

**Table S1.** Descriptive values for the correlation networks from different soil depths (forest floor (LFH) and mineral soil layers M1 (0-5 cm, M2 (5-15 cm), and M3 (15-30 cm)). The *Network* *Density* is a measure of how many correlations a community form is relative to the number of genera it is composed of. A higher density indicates that the genera have a high co-occurrence with other genera and may suggest more interactions and association per genus.

|  | LFH | M1 | M2 | M3 |
| --- | --- | --- | --- | --- |
| Number of genera | 96 | 105 | 100 | 64 |
| Bacteria genera (%) | 54.2 | 53.3 | 48 | 54.7 |
| Fungal genera (%) | 40.6 | 41.9 | 44 | 37.5 |
| Eukaryote genera (%) | 5.2 | 3.8 | 8 | 7.8 |
| Network Density | 0.015 | 0.024 | 0.016 | 0.023 |
| Proportion of positive  correlations (%) | 73.8 | 73.5 | 66.0 | 57 |
| Proportion of negative  correlations (%) | 26.2 | 26.5 | 34.0 | 43.0 |

**Table S2.** Wilcoxon signed-rank test on essential network topology measures. The test was performed between all pairs of soil depths, forest floor (LFH) and three mineral soil layers (M1 (0-5 cm, M2 (5-15 cm), and M3 (15-30 cm)) in birch stands at five locations in western Norway. P-values <0 .05 are marked in bold, and show the where two depths differ significantly. The *average degree* of a genus is the number of correlations it has with other genera. I.e. it is the number of connections (edges) formed by a node to other nodes. The *clustering coefficient* describes whether the network can be sectioned into clusters of groups of statistically associated organisms. The *Average path length* is the distance (counted as number of edges) between all pairs of associated genera (divided by the number of genera in the network). The *neighbourhood connectivity* is the average connectivity (correlations) of neighbours of a given node. I.e. nodes correlated to a given node can themselves be correlated to other nodes.

|  | LFH : M1 | LFH : M2 | LFH : M3 | M1 : M2 | M1 : M3 | M2 : M3 |
| --- | --- | --- | --- | --- | --- | --- |
| Average degree | **0.029** | 0.666 | 0.763 | 0.052 | 0.105 | 0.737 |
| Clustering coefficient | 0.118 | 0.233 | 0.262 | **0.001** | **0.008** | 0.824 |
| Average path length | **0.000** | **0.000** | 0.949 | **0.000** | **0.000** | **0.001** |
| Neighbourhood connectivity | **0.002** | 0.369 | 0.981 | **0.006** | **0.032** | 0.366 |

**Fig. S1.** A map showing five different sampling locations from native birch forest in western Norway. Within each location, three 144 m^2^ plots were selected.


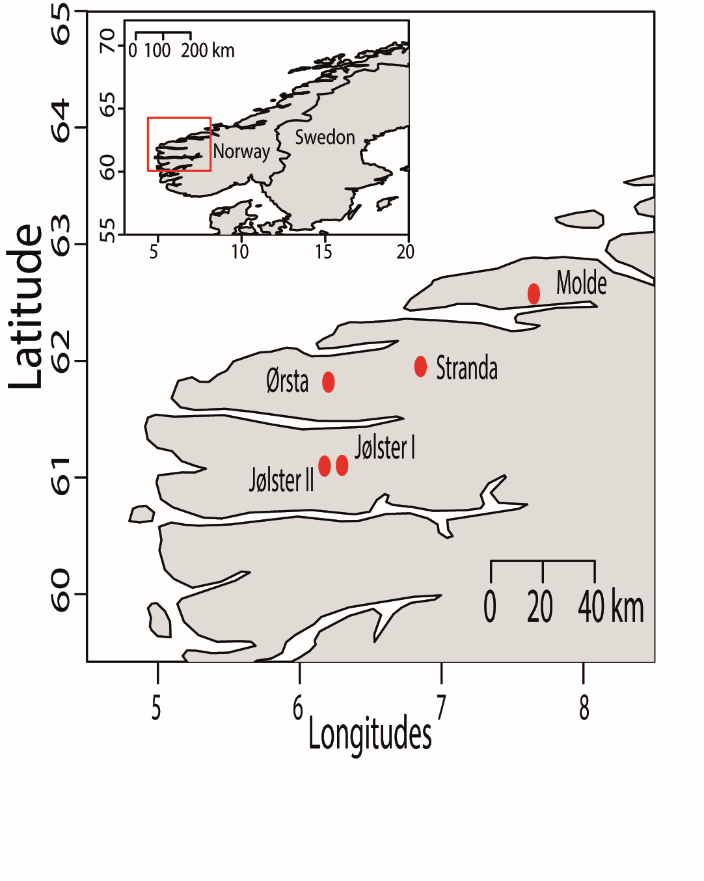


**Fig. S2.** Bar plots displaying distribution of reads per sample for bacteria (a), fungi (ITS: c; 18S: e), and micro-eukaryotes (g). Rank-abundance plot demonstrating the number of reads per OTU for bacteria (b), fungi (ITS: d; 18S: f), and micro-eukaryotes (h).


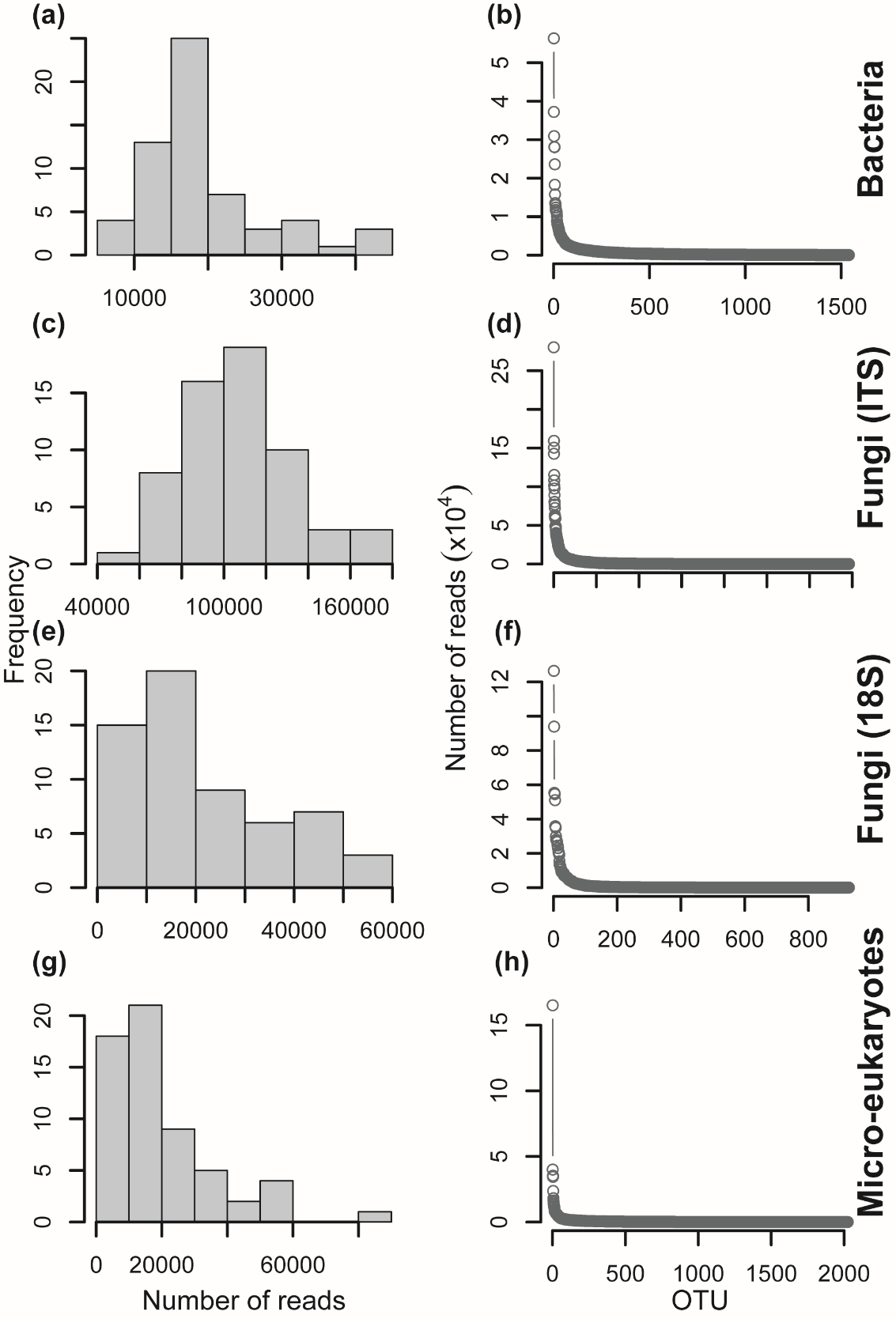


**Fig. S3.** Rarefaction curves describing number of observed Operational Taxonomic Units (OTUs) for bacteria (a), fungi (ITS: b; 18S: c), and micro-eukaryotes (d), as a function of sequencing reads numbers. Number of read mentioned in the panels indicates sequencing depth for data normalizations.


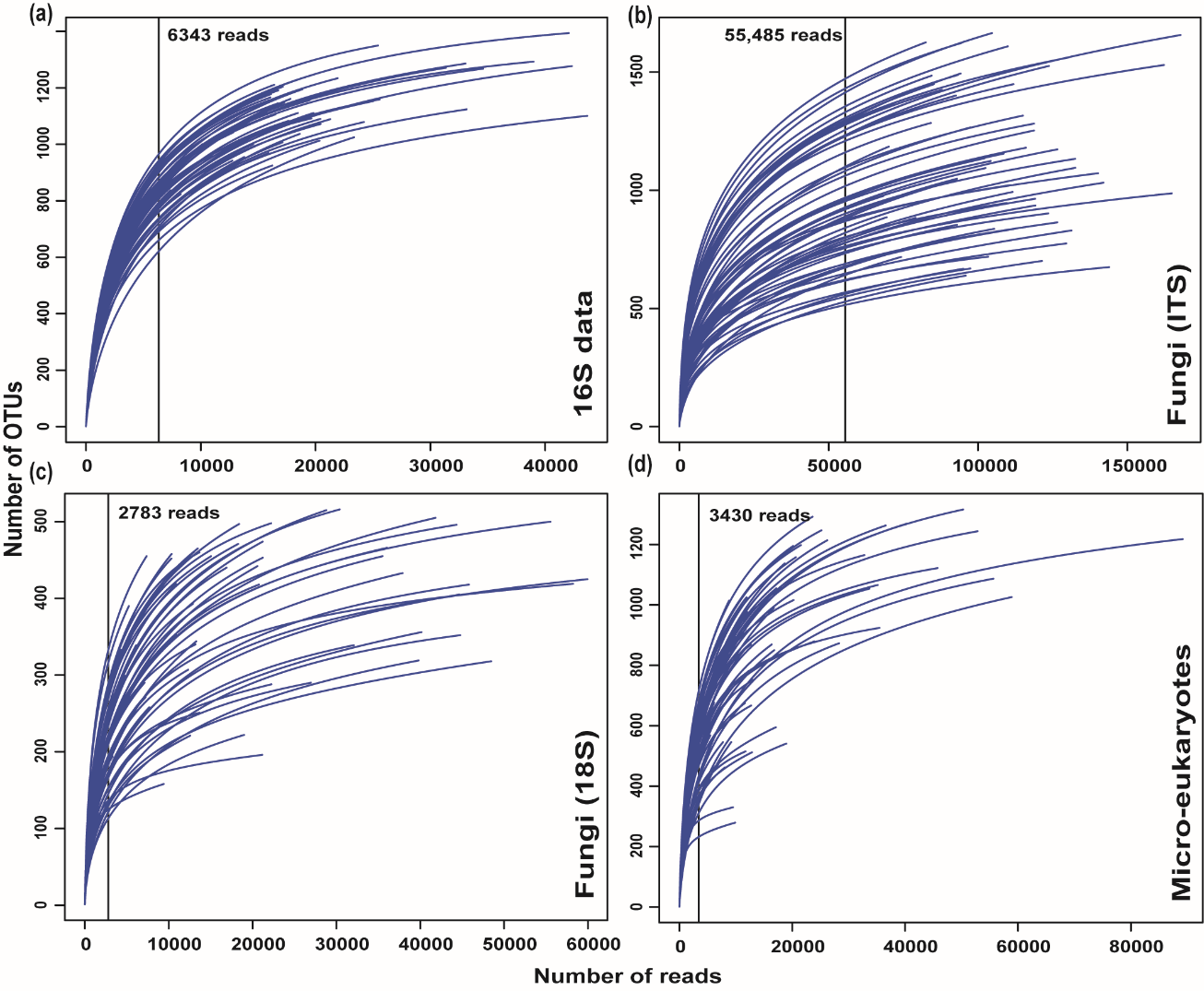


**Fig. S4.** Boxplots showing ergosterol (mg g^-1^ of soil) content, a “proxy” for fungal biomass, along a soil depth gradient (forest floor (LFH) and three mineral soil layers: 0-5 cm (M1), 5-15 cm (M2), and 15-30 cm (M3)).


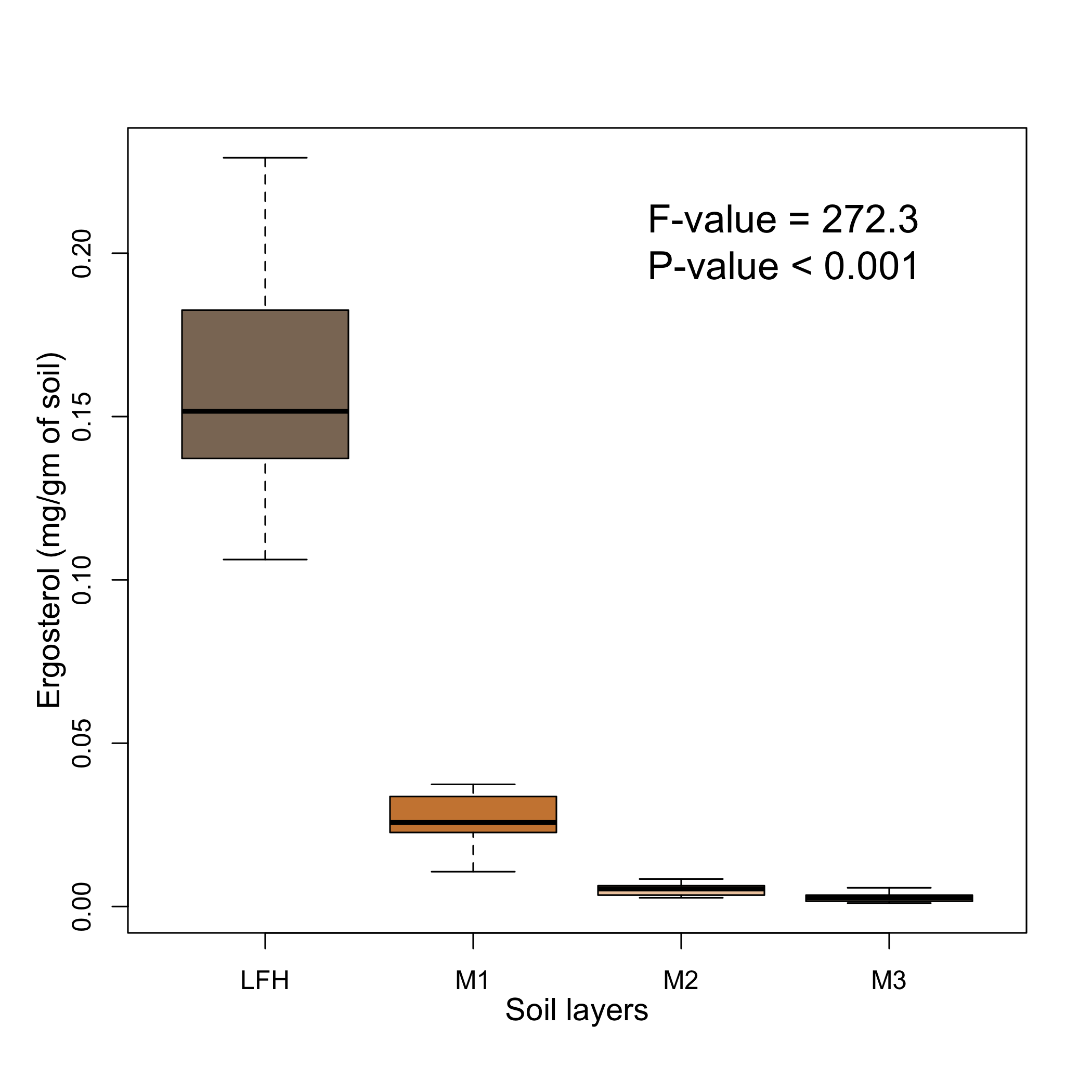


**Fig. S5.** Boxplots showing log-transformed data on changes in total C and N concentration and exchangeable nutrients with soil depth (forest floor (LFH) and three mineral soil layers: 0-5 cm (M1), 5-15 cm (M2), and 15-30 cm (M3)) in birch forest.


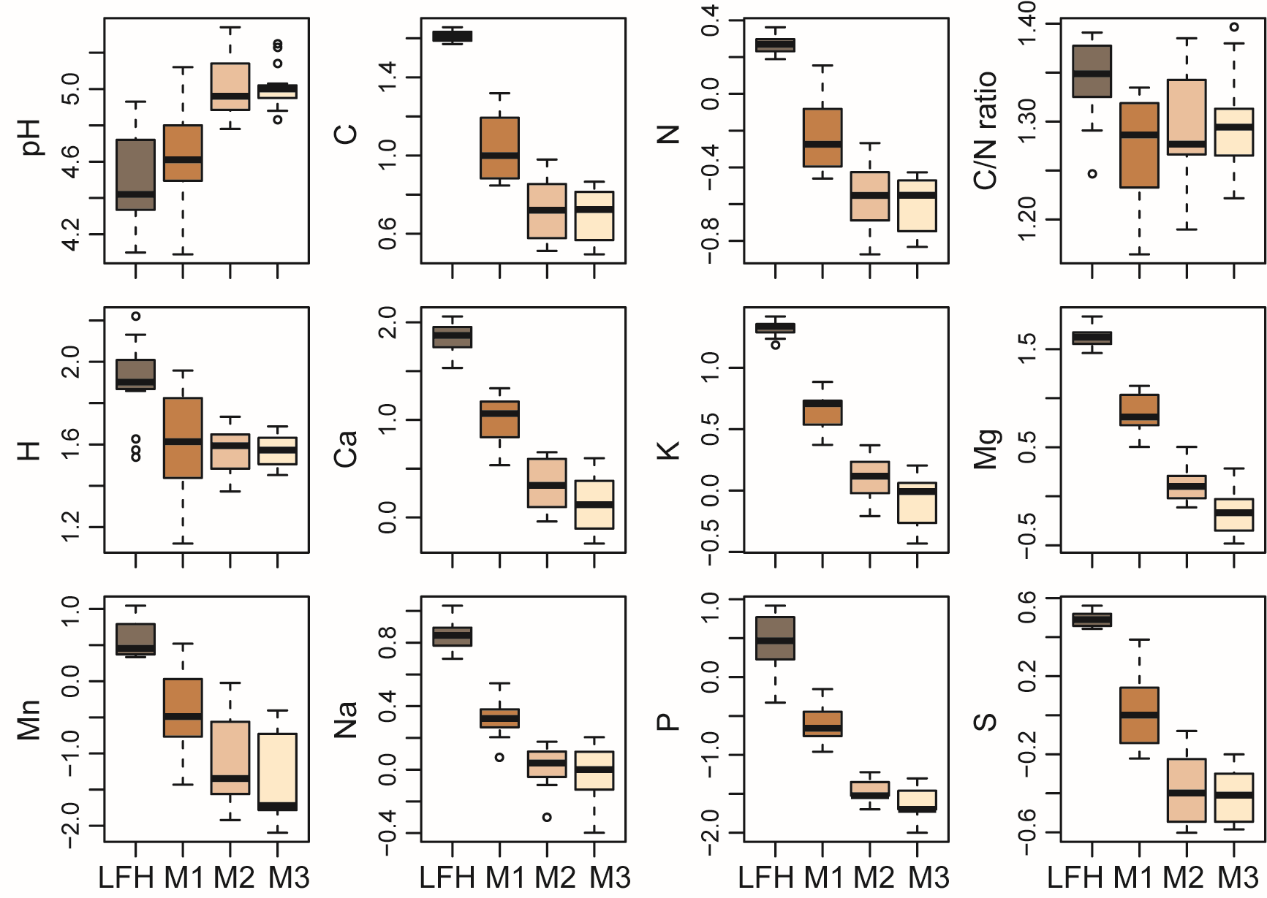


**Fig. S6.** A venn diagram showing unique and overlapping Operational Taxonomic Units (OTUs) for bacteria (a), fungi (ITS: b; 18S: c), and micro-eukaryotes (d) at different soil depths (forest floor (LFH), and three mineral soil layers: 0-5 cm (M1), 5-15 cm (M2), 15-30 cm (M3)). A total of 1540 bacterial, 4388 fungal (3461 ITS-based; 927 18S-based) and 2025 micro-eukaryotic OTUs were detected in this study using metabarcoding of 16S, ITS and 18S rRNA gene marker.


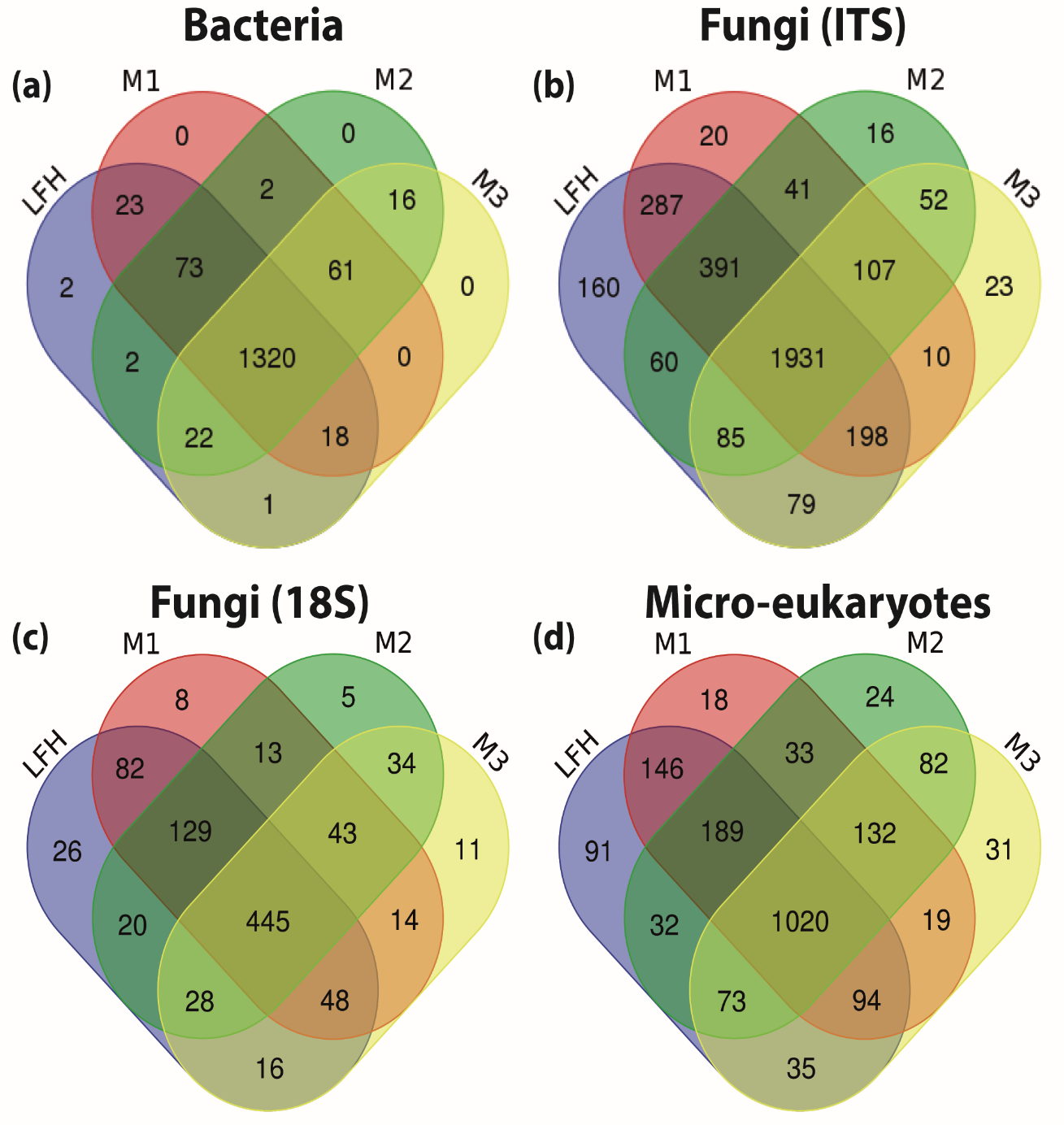


**Fig. S7** Inter-kingdom correlation patterns of bacterial, fungal and eukaryotic genera for forest floor layer “LFH”. The network is based on a SparCC correlation analysis for the genera recovered from forest floor (LFH). Nodes represents genera and are coloured according to taxonomic group: bacteria in orange, fungi in red, and micro-eukaryotes in blue. The size of a node is proportional to connection it forms with other nodes. Positive correlations (SparCC > 0.7, p < 0.05) are drawn as green edges and negative correlations (SparCC < 0.7, p < 0.05) are drawn as red edges. The thickness of the connection between two nodes is proportional to the value of correlation coefficients. The network in transparent grey is a reference network combining the correlations for all four depth layers.

**
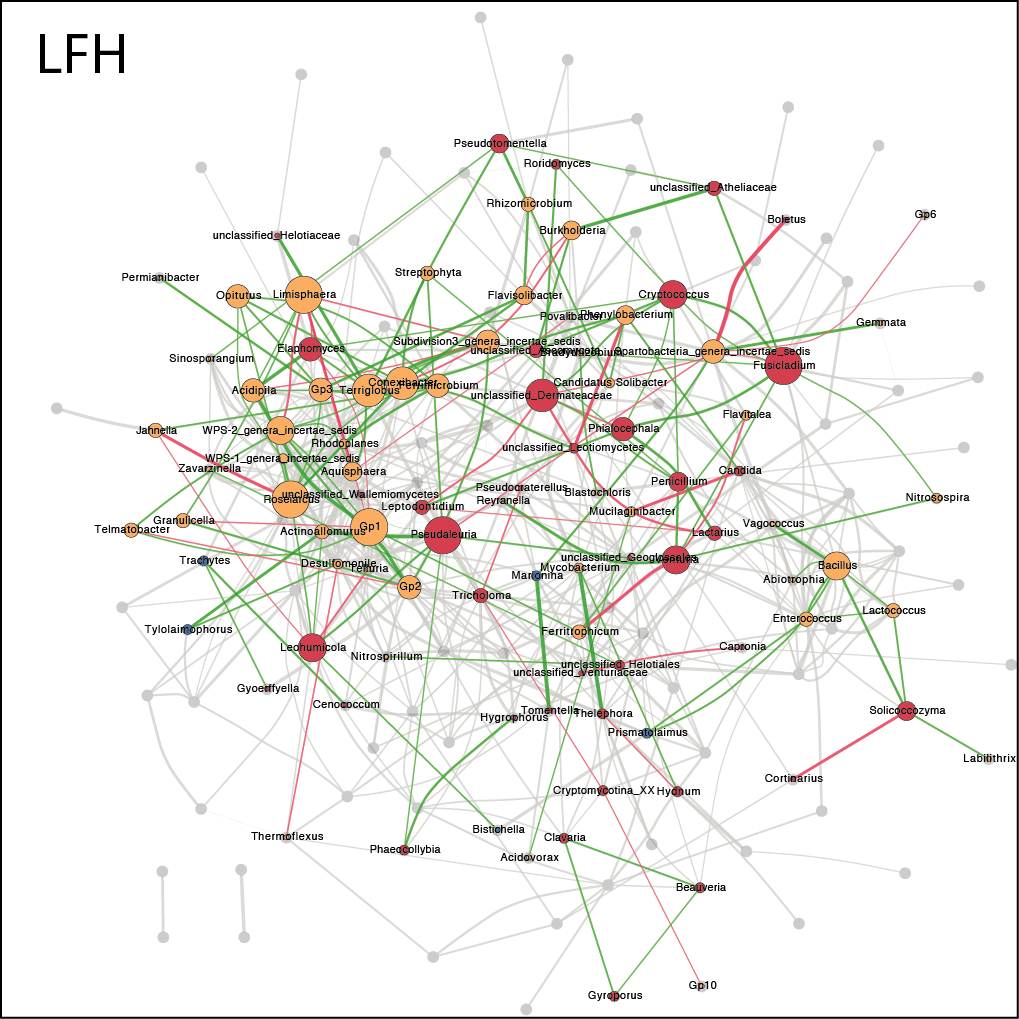
**

**Fig. S8.** Inter-kingdom correlation patterns of bacterial, fungal and eukaryotic genera for mineral soil layer “M1 (0-5 cm)”. The network is based on a SparCC correlation analysis for the genera recovered from M1 mineral soil layer. Nodes represents genera and are coloured according to taxonomic group: bacteria in orange, fungi in red, and micro-eukaryotes in blue. The size of a node is proportional to connection it forms with other nodes. Positive correlations (SparCC > 0.7, p < 0.05) are drawn as green edges and negative correlations (SparCC < 0.7, p < 0.05) are drawn as red edges. The thickness of the connection between two nodes is proportional to the value of correlation coefficients. The network in transparent grey is a reference network combining the correlations for all four depth layers.

**
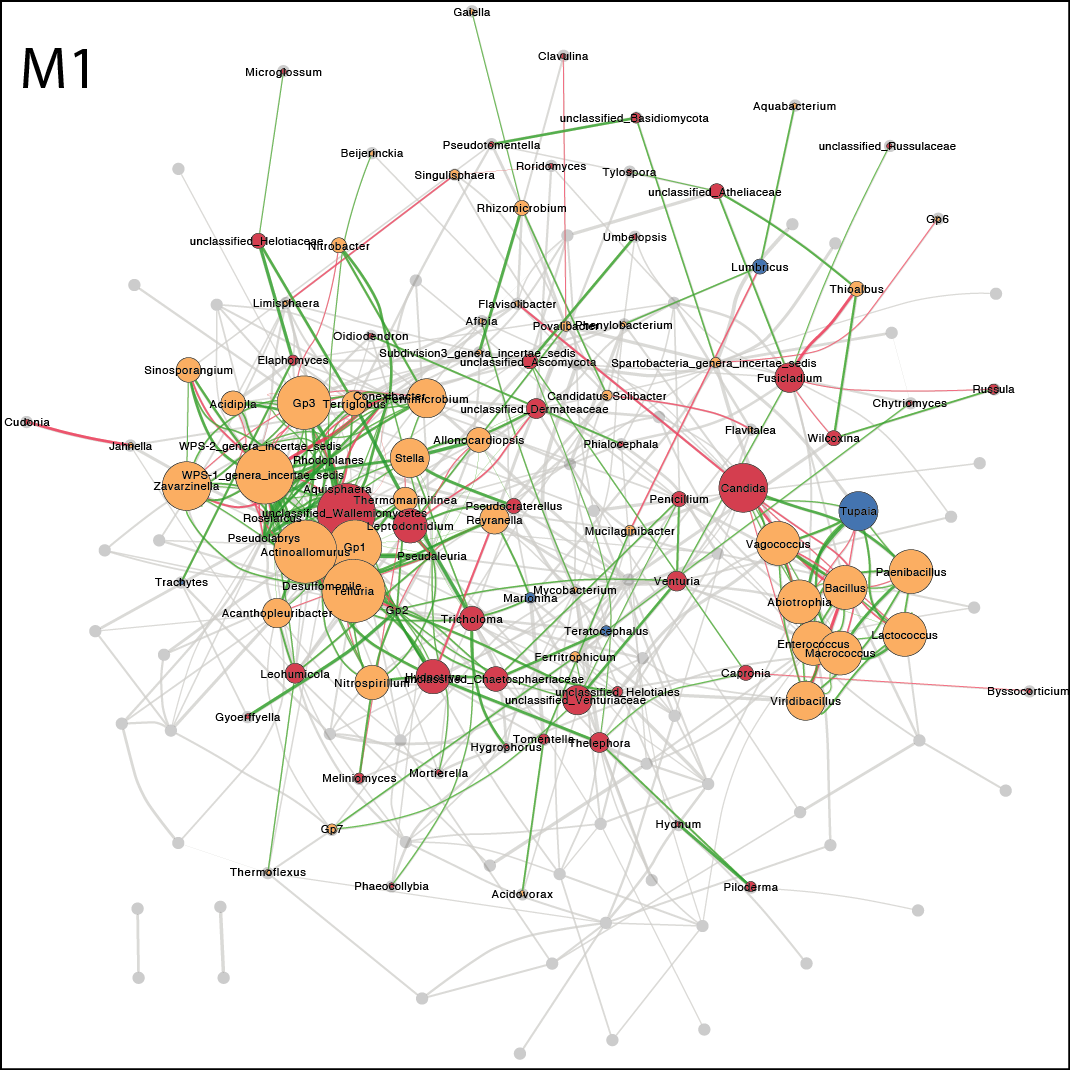
**

**Fig. 9** Inter-kingdom correlation patterns of bacterial, fungal and eukaryotic genera for mineral soil layer “M2 (5-15 cm)”. The network is based on a SparCC correlation analysis for the genera recovered from M2 mineral soil layer. Nodes represents genera and are coloured according to taxonomic group: bacteria in orange, fungi in red, and micro-eukaryotes in blue. The size of a node is proportional to connection it forms with other nodes. Positive correlations (SparCC > 0.7, p < 0.05) are drawn as green edges and negative correlations (SparCC < 0.7, p < 0.05) are drawn as red edges. The thickness of the connection between two nodes is proportional to the value of correlation coefficients. The network in transparent grey is a reference network combining the correlations for all four depth layers.

**
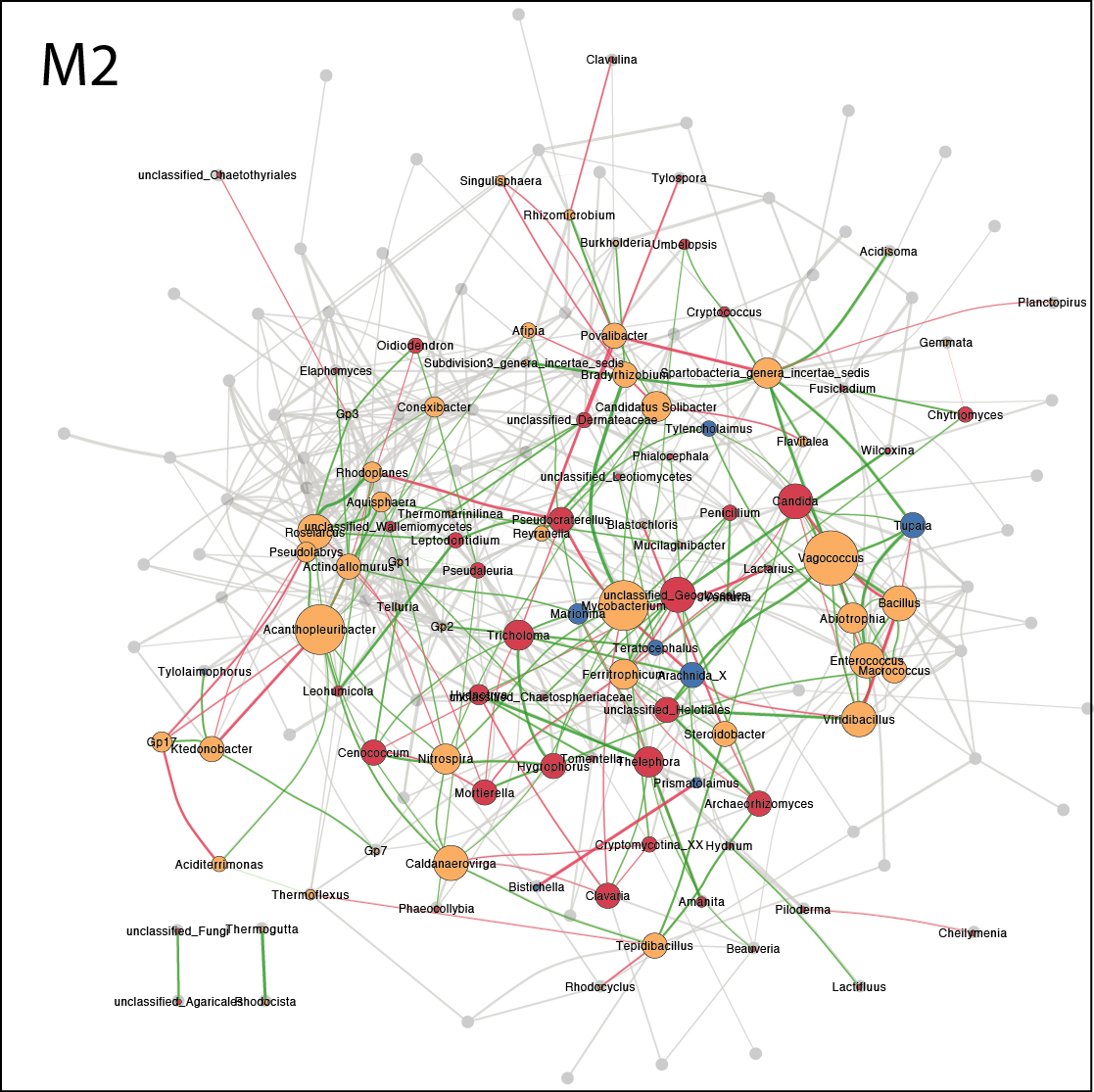
**

**Fig. S10** Inter-kingdom correlation patterns of bacterial, fungal and eukaryotic genera for mineral soil layer “M3 (15-30 cm)”. The network is based on a SparCC correlation analysis for the genera recovered from M2 mineral soil layer. Positive (Spearman’s ρ > 0.8) and significant (P < 0.01) co-occurrences are represented by green edges. Negative (Spearman’s ρ < 0.8) and significant (P < 0.01) co-exclusions are represented by red edges. The size of each node is proportional to the relative abundance of the genus; the thickness of each connection between two nodes (Ings, et al.) is proportional to the value of Spearman’s correlation coefficients. The nodes are coloured by microbial group (orange: bacteria, red: fungi, blue: micro-eukaryotes).

**
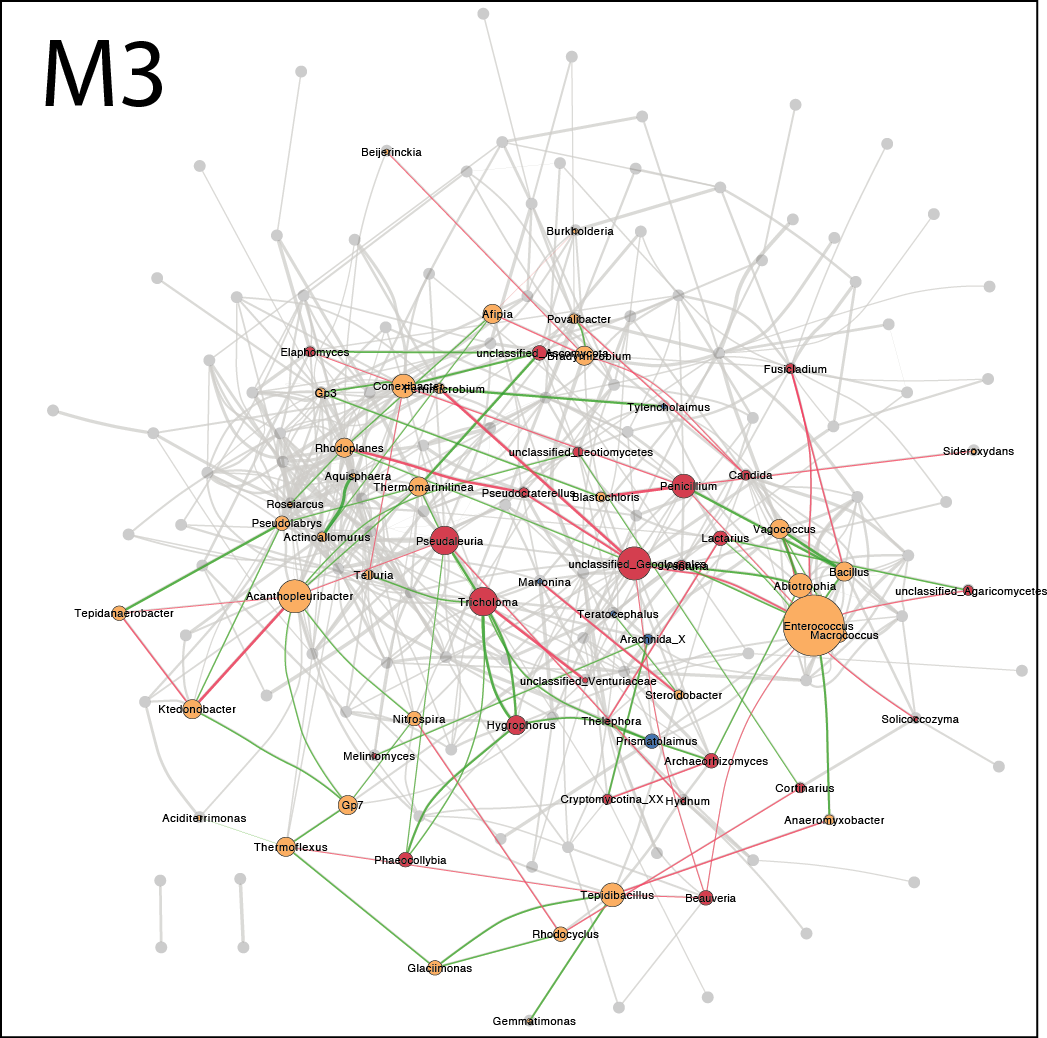
**

**Fig. S11** Venn diagram of genera with significant correlations with soil depth (forest floor (LFH) and three mineral soil layers: 0-5 cm (M1), 5-15 cm (M2), and 15-30 cm (M3). The genera unique for each depth as well as those common for all depths are listed in boxes. Taxonomic groups are marked within the boxes as (B) for bacteria (F) for fungi and (E) for micro-eukaryotes.

**
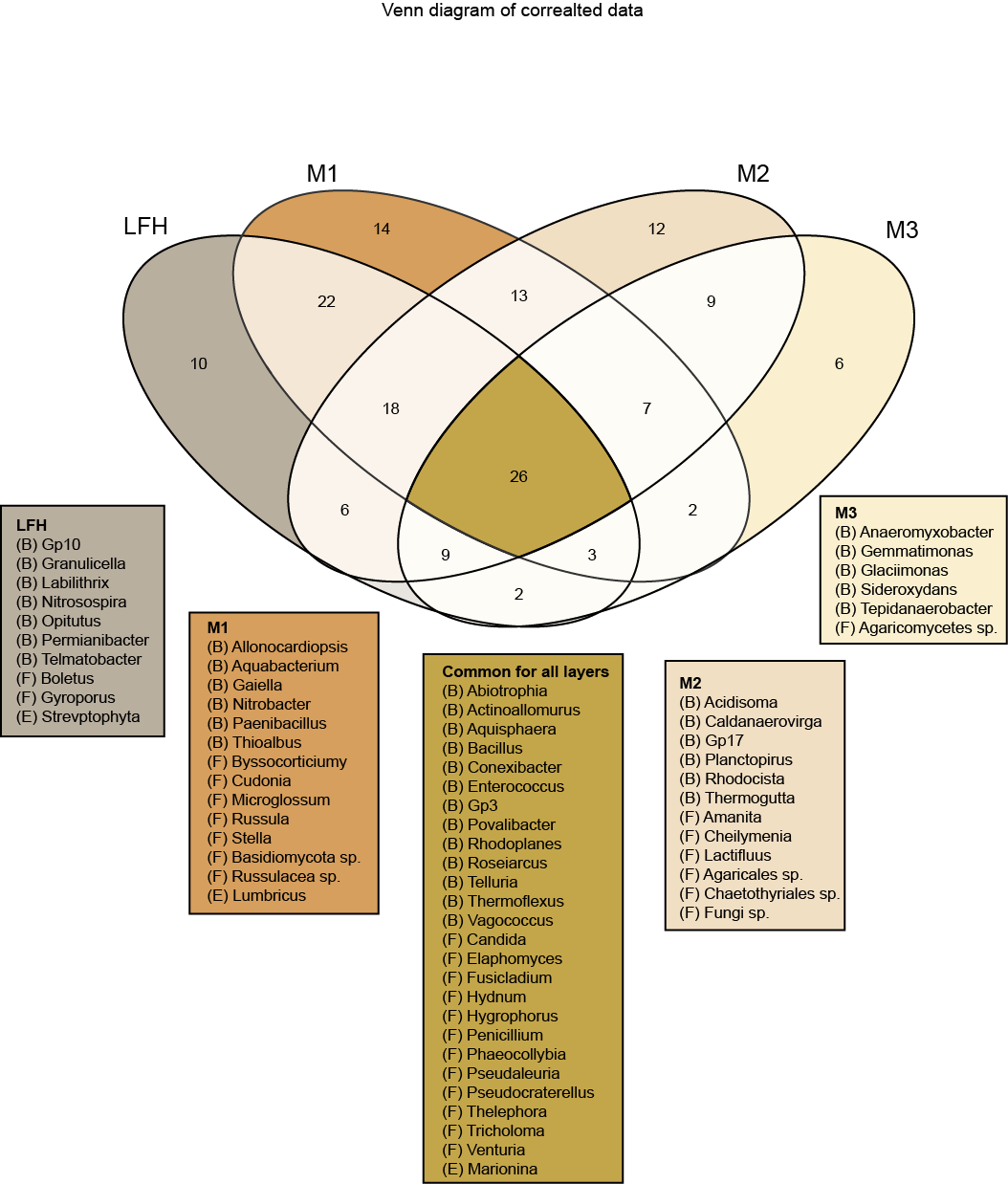
**

**References**

Caporaso JG, Lauber CL, Walters WA *et al.* Global patterns of 16S rRNA diversity at a depth of millions of sequences per sample. *Proceedings of the National Academy of Sciences* 2011;**108**: 4516-22.

Ihrmark K, Bödeker ITM, Cruz-Martinez K *et al.* New primers to amplify the fungal ITS2 region – evaluation by 454-sequencing of artificial and natural communities. *FEMS Microbiol Ecol* 2012;**82**: 666-77.

Ings TC, Montoya JM, Bascompte J *et al.* Review: Ecological networks – beyond food webs. *J Anim Ecol* 2009;**78**: 253-69.

Stoeck T, Bass D, Nebel M *et al.* Multiple marker parallel tag environmental DNA sequencing reveals a highly complex eukaryotic community in marine anoxic water. *Mol Ecol* 2010;**19**: 21-31.

White T, Bruns T, Lee S *et al.* Amplification and direct sequencing of fungal ribosomal RNA genes for phylogenetics. In: Innis M, Gelfand D, Shinsky J, White T (eds.) *PCR Protocols: A Guide to Methods and Applications*, DOI citeulike-article-id:671166. New York: Academic Press, San Diego, 1990, 315-22.
